# Sesquiterpenes from *Streptomyces qinglanensis* and Their Cytotoxic Activity

**DOI:** 10.3390/md21060361

**Published:** 2023-06-16

**Authors:** Cao Van Anh, Jong Soon Kang, Jeong-Wook Yang, Joo-Hee Kwon, Chang-Su Heo, Hwa-Sun Lee, Chan Hong Park, Hee Jae Shin

**Affiliations:** 1 Marine Natural Products Chemistry Laboratory, Korea Institute of Ocean Science and Technology, 385 Haeyang-ro, Yeongdo-gu, Busan 49111, Republic of Korea; caovananh12a1@gmail.com (C.V.A.); science30@kiost.ac.kr (C.-S.H.); hwasunlee@kiost.ac.kr (H.-S.L.); 2 Laboratory Animal Resource Center, Korea Research Institute of Bioscience and Biotechnology, 30 Yeongudanjiro, Cheongju 28116, Republic of Korea; kanjon@kribb.re.kr (J.S.K.); z7v8@kribb.re.kr (J.-W.Y.); juhee@kribb.re.kr (J.-H.K.); 3 Department of Marine Biotechnology, University of Science and Technology (UST), 217 Gajungro, Yuseong-gu, Daejeon 34113, Republic of Korea; 4 Dokdo Research Center, Korea Institute of Ocean Science and Technology, 48 Haeyanggwahak-gil, Jukbyeon-myeon, Uljin-gun 767-813, Gyeongsangbuk-do, Republic of Korea; chpark@kiost.ac.kr

**Keywords:** *Streptomyces* sp., sesquiterpenes, pentalenene, bolinane, cytotoxicity

## Abstract

Nine sesquiterpenes, including eight pentalenenes (**1**–**8**) and one bolinane derivative (**9**), were isolated from the culture broth of a marine-derived actinobacterium *Streptomyces qinglanensis* 213DD-006. Among them, **1**, **4**, **7**, and **9** were new compounds. Their planar structures were determined by spectroscopic methods (HRMS, 1D, and 2D NMR), and the absolute configuration was established by biosynthesis consideration and electronic-circular-dichroism (ECD) calculations. All the isolated compounds were screened for their cytotoxicity against six solid and seven blood cancer cell lines. Compounds **4**–**6** and **8** showed a moderate activity against all of the tested solid cell lines, with GI_50_ values ranging from 1.97 to 3.46 µM.

## 1. Introduction

The genus *Streptomyces* is renowned as the largest producer of secondary metabolites for the discovery of new antibiotics [1]. The genus is extremely important in biotechnology, producing approximately two-thirds of all antibiotics, as well as numerous chemical structures of medicinal and agricultural interest [2]. In addition to the production of antibiotics, members of this group of Gram-positive bacteria are also able to produce various classes of currently licensed drugs with other biomedical properties, such as doxorubicin as an anti-cancer [3], tacrolimus as an immunosuppressive [4], and avermectin as an antiparasitic drug [5]. The large diversity of structurally distinct bioactive compounds produced by over 900 species of *Streptomyces* can be attributed to their large modular biosynthetic gene clusters responsible for the production of many complex secondary metabolites [6,7]. Over the last few years, nearly 300 new compounds have been reported from this genus, and many of them showed promising activities [1]. Previous studies have conservatively estimated that the genus could produce another 150,000 antimicrobial natural products besides those currently known, suggesting that *Streptomyces* is far from an exhausted resource [8]. Therefore, marine *Streptomyces*-derived compounds are still one of the fascinating sources for new drug discovery.

Sesquiterpenes are one of the most abundant classes of secondary metabolites produced by all kingdoms of life [9]. Their identification is still increasing as a result of rising interest in marine biodiversity and the significance of natural products in drug discovery [10]. Reported sesquiterpenes are derived metabolically from about 300 distinct C15-hydrocarbon skeletons, and they display a broad range of biological activities such as anti-HIV, anticancer, antibacterial, antiviral, immunosuppressive, anti-inflammatory, insecticidal, and antifungal activities and have inspired research for druggable analogs [10]. The genus *Streptomyces* is also able to produce sesquiterpenes of various skeletons, such as eudesmane [11], pentalenene [12], and zizaene derivatives [13]. Therefore, further research can be conducted to discover new structures and biomedical applications of sesquiterpenes, especially in *Streptomyces*-derived compounds.

**Figure 1:**
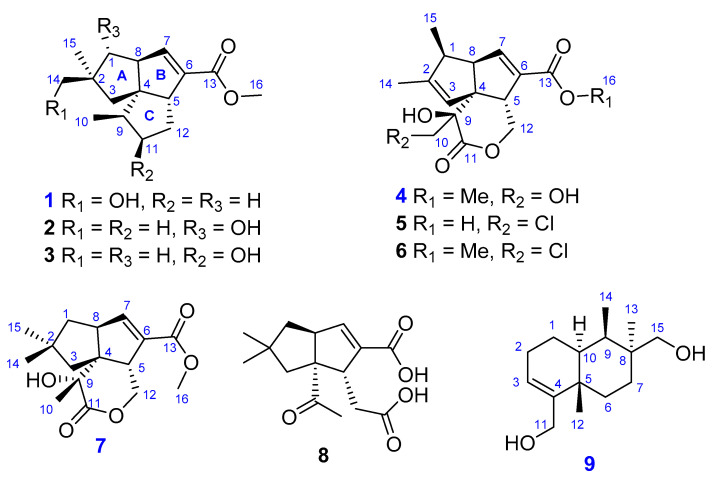
Structures of **1**–**9** isolated from *Streptomyces qinglanensis* 213DD-006.

In this study, *Streptomyces qinglanensis* 213DD-006 was isolated from a sediment sample collected off the coasts of Dokdo island, Republic of Korea. Interestingly, the small culture (1.0 L) extract from this strain showed significant growth inhibition against a panel of Gram-positive bacteria, and it also moderately inhibited the growth of certain Gram-negative bacteria. Furthermore, literature reviews have revealed that one of the major secondary metabolites of *Streptomyces qinglanensis* is enterocin, a polar polycyclic polyketide possessing a strong and broad anti-microbial activity [14]. Moreover, the ^1^H NMR spectrum of the non-polar fraction of the small culture extract from this strain showed certain unique peaks in olefinic and aliphatic regions, which were significantly different from those of enterocin. Therefore, based on the bioassay-guided and NMR-dereplication approaches, a large-scale fermentation was conducted for further study of the natural products (NPs) from the strain. Consequently, nine sesquiterpenes, including four new compounds (**1**, **4**, **7**, and **9**), were isolated from the strain (Figure 1). Herein, we describe the isolation, structure determination, and cytotoxic activity of these compounds. 

## 2. Results and Discussion

**Figure 2:**
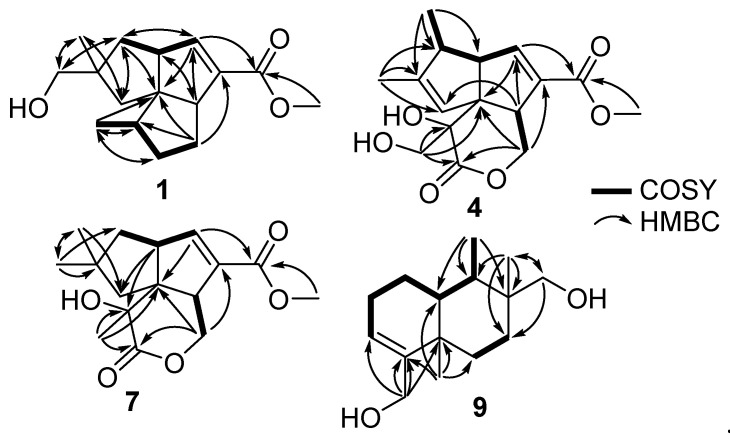
Key ^1^H-^1^H COSY and HMBC data of **1**, **4**, **7**, and **9**.

Compound **1** was isolated as a colorless solid. The molecular formula of **1** was determined as C_16_H_24_O_3_ by HRESIMS data (*m/z* 287.1624, calculated for C_16_H_24_O_3_Na, 287.1623), indicating that **1** was an isomer of the co-isolated compound, pentalenic acid methyl ester (**2**) [15]. The ^1^H NMR spectrum (Table 1) indicated the presence of an olefinic proton at *δ*_H_ 6.64 (dd, *J* = 2.2 and 1.5 Hz, H-7); a methoxy at *δ*_H_ 3.70 (s, H_3_-16); an oxygenated methylene at *δ*_H_ 3.29 (d, *J* = 3.9 Hz, H_2_-14); two methines at *δ*_H_ 3.02 (bd, *J* = 9.1 Hz, H-5) and 2.94 (ddd, *J* = 9.0, 5.8, and 2.7 Hz, H-8); two methyl groups at *δ*_H_ 1.04 (s, H_3_-15) and 0.97 (d, *J* = 7.1 Hz, H_3_-10); and other signals appearing in the region of *δ*_H_ 1.36–2.00 ppm. The ^13^C NMR spectrum, in combination with the gHSQC spectrum, revealed sixteen carbon signals, including a carbonyl at *δ*_C_ 167.5 (C-13); two olefinic carbons at *δ*_C_ 148.8 (C-7) and 138.2 (C-6); an oxygenated methylene *δ*_C_ 70.9 (C-14); a quaternary carbon at *δ*_C_ 65.5 (C-4); two methines at *δ*_C_ 60.0 (C-8) and 58.6 (C-5); a methoxy at *δ*_C_ 51.8 (C-16); and eight other carbons in the aliphatic region (Table 2). Two spin systems of **1** were determined by the continuous ^1^H-^1^H COSY correlations from H_3_-10 to H_2_-12 and from H_2_-1 to H-7 through H-8. An *α*,*β*-unsaturated carbonyl was inferred from the carbon data and confirmed by the HMBC correlation from H-7 to C-13. A methyl ester was identified by the strong HMBC correlation from H_3_-16 to C-13. The fact that CH_2_-14 bearing a hydroxy group was evidenced by the chemical shift values of H_2_-14 (*δ*_H_ 3.29) and C-14 (δ_C_ 70.9), as well as the HMBC correlations from H-1_a_,_b_, H-3_a_,_b_, and H_3_-15 to C-14. A pentalenene-type skeleton was proposed based on a detailed analysis of the HMBC data, and the planar structure of **1** was elucidated as shown in Figure 2.

**Figure 3:**
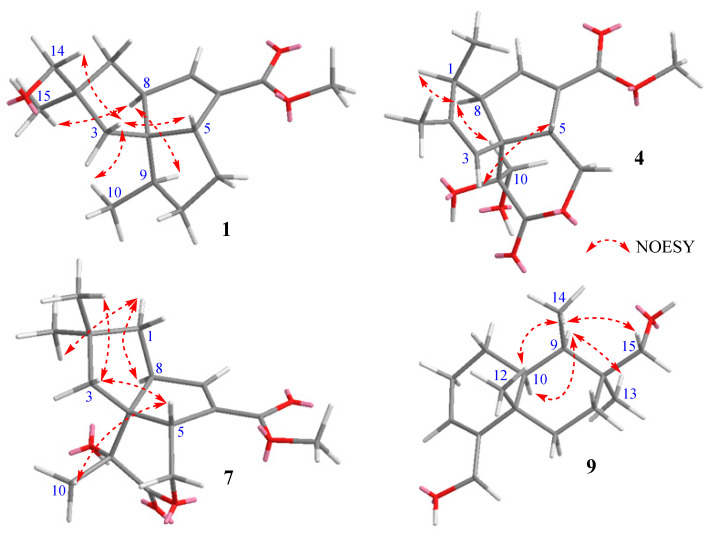
Key NOESY data of **1**, **4**, **7**, and **9**.

The relative configuration of **1** was determined by 2D NOESY experiments (Figure 3). The strong NOESY correlations of H-5/H_2_-14, H-5/H-3_b_, and H-3_b_/H_3_-10 indicated that H-5, H_3_-10, H-3_b_, and H_2_-14 had a co-facial relationship. There was no observed NOESY correlation between H-5 and H-8, and the strong NOESY correlations of H-8/H-9 and H-8/H_3_-15 determined that H-8, H-9, and H_3_-15 were located on the opposite face of the molecule. Hence, the relative configuration of **1** was determined to be the same as that of pentalenic acid methyl ester (**2**) [15]. Pentalenenes and pentalenolactones are a common group of sesquiterpenes isolated from the genus *Streptomyces* [12]. The structures of pentalenenes are quite rigid and composed of three fused cyclopentane rings with a double bond at the C-6 and C-7 positions of ring B [12,16]. Pentalenolactones are derived from pentalenenes via the oxidation of ring C to form a lactone bond between C-11 and C-12 [16,17]. The biosynthesis of pentalenenes and pentalenolactones from a farnesyl unit by a pentalenene synthase has been very well studied [18]. The structures of these sesquiterpenes have also been confirmed by many total-synthesis studies [19]. Previous studies have revealed that H-8 and C-9 and C-12 have an *α*-orientation and H-5 has a *β*-orientation in all of the reported pentalenene and pentalenolactone derivatives. Therefore, the absolute configuration of **1** was determined as 2*S*, 4*R*, 5*R*, 8*S*, and 9*R* by considering its biosynthetic correlation with other pentalenene-type sesquiterpenes. Thus, **1** is a new analog of the co-isolated compound, pentalenic acid methyl ester (**2**), and is named pentalenomycin A. 

Compound **4** was isolated as a colorless solid with the molecular formula of C_16_H_20_O_6_ by HRESIMS data (*m/z* 331.1158, calcd for C_16_H_20_O_6_Na, 331.1158). The ^1^H NMR data of **4** were almost identical to those of pentalenolactone I methyl ester (**6**); the significant difference between **4** and **6** was the chemical shift of C-10 (*δ*_C-10_ was 63.4 ppm for **4**, and 46.8 ppm for **6**, Figure S8) [16], indicating that the chlorine atom attached to C-10 of **6** was changed to an oxygen atom in **4**, which was further confirmed by HRESIMS data. The strong NOESY correlations from H-8 to H-1 and H-10_a_ indicated that H-1, H-8, and H-10_a_ were located on the same face of the molecule. The lack of a NOESY correlation from H-8 to H-5 revealed that H-5 was located on the opposite face from H-8. Thus, the relative configuration of **4** was determined to be the same as that of pentalenolactone I methyl ester (**6**). The absolute configuration of **4** was proposed to be the same as that of **6** by considering the biosynthetic relationship of **4** and **6.** Hence, **4** was elucidated as a new derivative of **6**, as shown in Figure 1, and was given the trivial name pentalenomycin B.

Compound **7** was isolated as a colorless solid. The molecular formula of **7** was determined as C_16_H_22_O_5_ by HRESIMS data (*m/z* 317.1364, calcd for C_16_H_20_O_5_Na, 317.1365) with one more degree of unsaturation than that of pentalenolactone F methyl ester and one more singlet methyl appearing at *δ*_H_ 1.56 ppm (H_3_-10), indicating that **7** was a new analog of pentalenolactone F methyl ester with a ring-opening type instead of an epoxide group at C-9 and C-10 [20]. The strong NOESY correlations from H-5 to H_3_-10 indicated that H-5 and H_3_-10 were located on the same face of the molecule. There was no NOESY correlation from H-8 to H-5, indicating that H-8 was located on the opposite face from H-5. Thus, the relative configuration of **7** was determined to be the same as that of pentalenolactone F methyl ester. The absolute configuration of **7** was proposed to be the same as that of pentalenolactone F methyl ester, whose absolute stereochemistry was determined by X-ray crystallography [20], by considering their biosynthetic relationship and by comparing NMR data. Hence, the structure of **7** was elucidated, as shown in Figure 1, and was given the trivial name pentalenomycin C. 

**Figure 4:**
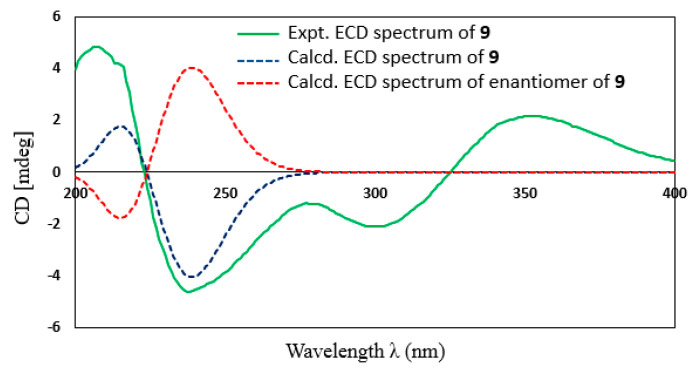
Experimental and calculated ECD spectra of **9**.

Compound **9** was isolated as a colorless solid. The molecular formula of **9** was determined as C_15_H_26_O_2_, requiring three degrees of unsaturation. The ^13^C in combination with HSQC NMR data revealed signals of 15 carbons, 2 of which were in the olefinic region (*δ*_C_ 148.0 and 122.6), which accounted for one out of three degrees of unsaturation, indicating that **9** was a bicyclic sesquiterpene. The ^1^H and ^13^C NMR data of **9** were quite similar to those of bolinaquinone (Figure S25), except for the lack of signals for a quinone ring [21]. Further detailed analysis of HMBC and ^1^H-^1^H COSY data (Figures S29 and S30) confirmed the planar structure of **9** as shown in Figure 1. The relative configuration of **9** was determined to be the same as that of bolinaquinone by 1D selective NOESY experiments (Figures S31–S33). Indeed, the strong NOESY correlations from H_3_-14 to H-15_a,b_, and H_3_-12 indicated that H_3_-14, H-15_a,b_, and H_3_-12 have a co-facial relationship and those of H_3_-13/H-9 and H_3_-13/H-10 indicated that H_3_-13, H-9, and H-10 were located on the opposite side of the molecule. Further, the theoretical ECD spectra and specific optical rotation values of **9** and its enantiomer were calculated (Figure 4). The experimental ECD spectrum and the sign of optical rotation of **9** (${\left[\alpha \right]}_{D}^{20}$ + 75) were matched with its theoretical ECD spectrum and the calculated optical rotation value ${\left[\alpha \right]}_{D}^{20}$ + 22.7. The structure of **9** was therefore determined as depicted and named bolinane A. A literature review revealed that several hybrid sesquiterpenes with a quinone ring (bolinaquinones) have been isolated from marine sponges; however, the absolute configuration of bolinaquinones has not yet been reported [22]. To the best of our knowledge, **9** is the first example of a bolinane-type sesquiterpene (not hybrid), and the absolute configuration of bolinane-type sesquiterpenes was determined for the first time in this study.

The structures of the known compounds were determined as pentalenic acid methyl ester (**2**) [15], methyl (-)-11*β*-hydroxy-1-deoxypentalenate (**3**) [17], pentalenolactone I (**5**) [16,23], pentalenolactone I methyl ester (**6**) [23], and (1*R*,3a*S*,6a*R*)-6a-acetyl-1-(carboxymethyl)-5,5-dimethyl-1,3a,4,5,6,6a-hexahydropentalene-2-carboxylic acid (**8**) [24] by comparison of their spectroscopic data with those reported in the literature.

Each of the isolated compounds was evaluated for their cytotoxicity against six solid cancer cell lines (PC-3 (prostate), NCI-H23 (lung), HCT-15 (colon), NUGC-3 (stomach), ACHN (renal), and MDA-MB-231 (breast); see Table 3). Compounds **4**–**6** and **8** demonstrated a moderate activity against all of the tested cell lines, with GI_50_ values ranging from 1.97 to 3.46 µM. Furthermore, all of the compounds were also screened for their cytotoxic activity against seven blood cancer cell lines (HL-60 (acute myelogenous leukemia, AML), Raji (Burkitt’s lymphoma), K562 (chronic myelogenous leukemia, CML), RPMI-8402 (T cell acute lymphocytic leukemia, T-ALL), NALM6 (B cell acute lymphocytic leukemia, B-ALL), U266 (multiple myeloma), WSU-DLCL2 (diffuse large B cell lymphoma, DLBCL); see Table 4). Compounds **4**, **6**, and **8** showed selective but weak activities against several cell lines, as shown in Table 4. Other compounds did not demonstrate significant activities (GI_50_ > 30 µM). From the activity results, it is noteworthy that the tested compounds demonstrated more potent activity against solid cancer than against blood cancer cell lines, and pentalenolactones (**4**–**6**) showed a stronger cytotoxicity than pentalenene sesquiterpenes (**1**–**3**), indicating that the lactone ring (ring C) may be important for the bioactivities of pentalenolactones. However, further studies are needed to clarify this hypothesis and the mode of action of the isolated compounds.

## 3. Materials and Methods

### 3.1. General Experimental Procedures

The 1D and 2D NMR spectra were recorded utilizing a Bruker 600 MHz spectrometer (Bruker BioSpin GmbH, Rheinstetten, Germany). UV–Vis spectra were measured by a Shimadzu UV-1650PC spectrophotometer (Shimadzu Corporation, Kyoto, Japan). IR spectra were obtained by a JASCO FT/IR-4100 spectrophotometer (JASCO Corporation, Tokyo, Japan). High-resolution ESIMS experiments were conducted with a hybrid ion trap time-of-flight mass spectrometer (Shimadzu LC/MS-IT-TOF, Kyoto, Japan). HPLC was conducted using a PrimeLine Binary pump (Analytical Scientific Instruments, Inc., El Sobrante, CA, USA) and a RI-101 detector (Shoko Scientific Co., Ltd., Yokohama, Japan). Semi-preparative HPLC was performed by an ODS column (YMC-Pack-ODS-A, 250 × 10 mm i.d., 5 µM, Kyoto, Japan). Optical rotations were obtained by a Rudolph Research Analytical Autopol III polarimeter (Rudolph Research Analytical, Hackettstown, NJ, USA). All solvents were either HPLC grade or distilled prior to use. Mass culture was conducted using a 100-L fermenter (Fermentec Co., Ltd., Cheongju, Republic of Korea).

### 3.2. Isolation of the Microorganisms from Marine Sediment Samples

Marine sediment samples were collected offshore of Dokdo Island, Republic of Korea, during expeditions in March 2021. A grab sampler was used to collect the sediment samples at a depth of approximately 200 m below the water’s surface. After collection, the sediments were placed in sterile 50-mL conical tubes and stored at 5 °C upon return to the laboratory. Since *Actinomycetes* are spore-forming bacteria, they can survive in severe conditions with high temperatures because of their spore formation. Hence, non-spore-forming unwanted microorganisms were eliminated by selective heating pretreatment. A 1.0 g amount of each collected sample was put on a sterile plate and maintained in a dry oven at 60 °C for 30 min. The samples were then serially diluted to 10^−1^, 10^−2^, and 10^−3^ using sterile seawater, and each aliquot (100 µL) was spread on actinomycetes isolation agar (AIA), humic acid–vitamin agar (HV), and Bennett’s agar (BN) media. The plates were incubated at 28 °C for 1~4 weeks in a BOD (bio-oxygen demand) incubator until colonies became visible. Observed colonies were selected and transferred onto new BN agar plates. The purification process was repeated several times until single pure colonies were obtained. 

### 3.3. Isolation and Identification of the Strain 213DD-006

The strain 213DD-006 was isolated from actinomycetes isolation agar after incubation for 10 days and was identified as *Streptomyces qinglanensis* according to morphological characteristics and results of 16S-rRNA gene sequence analysis by Macrogen Inc. (Seoul, Republic of Korea). The sequence of 213DD-006 was submitted to GenBank under accession number OQ457699.

### 3.4. Fermentation of the Strain 213DD-006 and Extraction and Isolation of Metabolites

The seed and mass cultures of the strain 213DD-006 were conducted using Bennett’s medium. A single colony of the strain from an agar plate was aseptically transferred to a 2.0-L flask filled with 1.0 L of BN broth. The strain was incubated at 28 °C for 7 days on a rotary shaker at 140 rpm and then the culture broth was inoculated into a 100-L fermenter filled with 70 L of BN broth. The mass culture was carried out at 28 °C for 12 days and then harvested. The culture was separated into supernatant and mycelium by continuous centrifugation at 60,000 rpm, and the supernatant was extracted twice with an equal volume of EtOAc (70 L × 2). The EtOAc layer was evaporated under reduced pressure to obtain a crude extract (8.0 g). The extract was then fractionated into 15 fractions (fractions 1 to 15) by vacuum liquid chromatography on an ODS column using a stepwise elution with 3 × 300 mL each of 20%, 40%, 60%, and 80% MeOH in H_2_O and 100% MeOH. The F7 fraction was subjected to a semi-preparative HPLC (YMC-Pack ODS-A, 250 × 10 mm i.d., 5 μm, flow rate 2.0 mL/min) with an isocratic elution of 46% MeOH in H_2_O to obtain compounds **5** (3.0 mg, *t_R_* = 44 min), **8** (2.5 mg, *t_R_* = 50 min), and **4** (2.0 mg, *t_R_* = 54 min). The F8 fraction was purified using a semi-preparative HPLC (YMC-PackODS-A, 250 × 10 mm i.d., 5 μm, flow rate 2.0 mL/min) with an isocratic elution of 57% MeOH in H_2_O to yield compounds **6** (3.5 mg, *t_R_* = 42 min), **7** (2.0 mg, *t_R_* = 34 min), and **9** (3.0 mg, *t_R_* = 31 min). Compounds **1** (2.0 mg, *t_R_* = 28 min), **2** (3.3 mg, *t_R_* = 30 min), and **3** (4.2 mg, *t_R_* = 26 min) were isolated from the F10 fraction using a semi-preparative HPLC (YMC-PackODS-A, 250 × 10 mm i.d., 5 μm, flow rate 2.0 mL/min) with an isocratic elution of 70% MeOH in H_2_O. 

Pentalenomycin A (**1**): colorless solid, ${\left[\alpha \right]}_{D}^{20}$ − 30.5 (*c* 0.3, MeOH); UV (MeOH) *λ*_max_ (log *ε*) 227 (3.35) nm; IR *ν*_max_ 3423, 2946, 2865, 1706, 1625, 1441, 1246, 1031 cm^−1^; HRESIMS *m/z* 287.1624, calculated for C_16_H_24_O_3_Na, 287.1623; for ^1^H NMR (CD_3_OD, 600 MHz), see Table 1; for ^13^C NMR (CD_3_OD, 150 MHz), see Table 2.

Pentalenomycin B (**4**): colorless solid, ${\left[\alpha \right]}_{D}^{20}$ − 40.3 (*c* 0.1, MeOH); UV (MeOH) *λ*_max_ (log *ε*) 205 (3.43) nm; IR *ν*_max_ 3477, 2950, 1713, 1643, 1453, 1356, 1265, 1144, 1024 cm^−1^; HRESIMS *m/z* 331.1158, calculated for C_16_H_20_O_6_Na, 331.1158; for ^1^H NMR (CD_3_OD, 600 MHz), see Table 1; for ^13^C NMR (CD_3_OD, 150 MHz), see Table 2.

Pentalenomycin C (**7**): colorless solid, ${\left[\alpha \right]}_{D}^{20}$ − 36.7 (*c* 0.1, MeOH); UV (MeOH) *λ*_max_ (log *ε*) 225 (3.26) nm; IR *ν*_max_ 3494, 2946, 2858, 1713, 1639, 1453, 1349, 1243, 1141 cm^−1^; HRESIMS *m/z* 317.1364, calculated for C_16_H_20_O_5_Na, 317.1365; for ^1^H NMR (CD_3_OD, 600 MHz), see Table 1, for ^13^C NMR (CD_3_OD, 150 MHz), see Table 2.

Bolinane A (**9**): colorless solid, ${\left[\alpha \right]}_{D}^{20}$ + 75.0 (*c* 0.1, MeOH); UV (MeOH) *λ*_max_ (log *ε*) 206 (3.85) nm; IR *ν*_max_ 3349, 2925, 2869, 1643, 1455, 1377, 1003 cm^−1^; HRESIMS *m/z* 261.1829, calculated for C_15_H_26_O_2_Na, 261.1830; for ^1^H NMR (CD_3_OD, 600 MHz), see Table 1; for ^13^C NMR (CD_3_OD, 150 MHz), see Table 2.

### 3.5. Computational Details

Preliminary conformational searches were performed using molecular mechanics force-field (MMFF94s) calculations with a search limit of 3.0 kcal/mol in the CONFLEX version 8.0 program (CONFLEX Corporation). The low-energy conformers accounting for more than 1% were further optimized using the density functional theory (DFT) method at the B3LYP/6-31 + G(d,p) level. The theoretical ECD spectra and optical rotation values (ORD) were calculated by Gaussian 16 software using the time-dependent DFT (TD-DFT) method at the B3LYP/6-31 + G (d,p) level in MeOH with the polarizable continuum model (PCM) using the integral equation formalism variant (IEFPCM) model. The Boltzmann distribution of each conformer was calculated and relocated based on their Hartree energy, and the final calculated ECD spectra and optical rotation values (ORD) were obtained by averaging the ECD spectrum and the optical rotation value of each conformer according to their Boltzmann distributions.

### 3.6. Cytotoxicity Assay

The cytotoxic activities of **1**–**9** against adherent cells and suspension cells were conducted by SRB (sulforhodamine B) assay [25] and CellTiter-Glo assay [26], respectively, as previously described. One-way ANOVA followed by Dunnett’s *t*-test was used for statistical analysis, and the GI_50_ values were determined by the software of GraphPad Prism 8 (GraphPad Software Inc., San Diego, CA, USA). Cancer cell lines were purchased from the Japanese Cancer Research Resources Bank (JCRB) (NUGC-3, JCRB Cell Bank/Cat. # JCRB0822), the DSMZ-German Collection of Microorganisms and Cell Cultures (RPMI-8402, DSMZ/Cat # ACC 290; WSU-DLCL2, DSMZ/Cat # ACC 575), and the American Type Culture Collection (ATCC) (PC-3, ATCC/Cat. # CRL-1435; MDA-MB-231, ATCC/Cat. # HTB-26; ACHN, ATCC/Cat. # CRL-1611; NCI-H23, ATCC/Cat. # CRL-5800; HCT-15, ATCC/Cat. # CCL-225; HL-60, ATCC/Cat. # CCL-240; Raji, ATCC/Cat # CCL-86; K562, ATCC/Cat # CCL-243; NALM6, ATCC/Cat # CRL-3273; U266, ATCC/Cat # TIB-196).

## 4. Conclusions

In conclusion, we have isolated nine sesquiterpenes, including three new pentalenenes and a new bolinane derivative. Compound **9** is the first example of bolinane sesquiterpenes isolated from a natural source. The structures of the isolated compounds were established by spectroscopic methods, comparisons with data reported in the literature, and ECD calculations. All compounds were tested for six solid and seven blood cancer cell lines. Compounds **4**–**6** and **8** showed a moderate activity against all solid cancer cell lines, with GI_50_ values of 1.97–3.46 µM. Further studies should be conducted to understand the mode of action of the active compounds. The results of this study have expanded the chemical and biological diversities of naturally occurring sesquiterpenes from the genus *Streptomyces*.

## Figures and Tables

**Table 1:** ^1^H NMR data for **1**, **4**, **7**, and **9**.

	1	4	7	9
Pos.	*δ*_H_, Mult (*J* in Hz)	*δ*_H,_ Mult (*J* in Hz)	*δ*_H,_ Mult (*J* in Hz)	*δ*_H,_ Mult (*J* in Hz)
1	1.68, dd (13.0, 8.9) 1.42 (13.2, 6.0)	2.85, m	1.75, dd (12.9, 10.2) 1.56, ovl	1.82, m 1.25, dd (11.2, 6.6)
2				2.15, m
3	1.76, d (13.5), H-3_a_ 1.58, d (13.6), H-3_b_	5.41, brs	2.16, d (13.4), H-3_a_ 1.69, d (13.3), H-3_b_	5.53, s
5	3.02, d (9.1)	3.12, m	3.27, m	
6				1.57, m
7	6.64, dd (2.2, 1.5)	6.78, t (1.9)	6.81, t (2.1)	1.49, m 1.07, ovl
8	2.94, ddd (9.0, 5.8, 2.7)	3.24, dt (8.5, 2.9)	3.34, ovl	
9	1.91, m			1.53, m
10	0.97, d (7.1)	3.90, d (11.5) 3.44, d (11.5)	1.56, s	1.86, m
11	1.64, m 1.36, m			4.02, m
12	2.00, m 1.40, ovl	5.22, dd (11.2, 5.3) 4.39, dd (11.2, 0.6)	4.70, dd (12.4, 5.0) 4.56, dd (12.4, 1.9)	1.04, s
13				1.08, s
14	3.29, d (3.9)	1.68, t (1.30)	0.99, s	0.92, d (7.6)
15	1.04, s	1.03, d (7.40)	1.06, s	3.46, d (10.7) 3.18, d (10.7)
16	3.70, s	3.75, s	3.75, s	

**Table 2:** ^13^C NMR data for **1**, **4**, **7**, and **9**.

	1	4	7	9
Pos.	*δ*_C_, Type	*δ*_C_, Type	*δ*_C_, Type	*δ*_C_, Type
1	41.8, CH_2_	45.9, CH	45.6, CH_2_	25.5, CH_2_
2	47.1, C	146.8, C	41.4, C	27.4, CH_2_
3	44.3, CH_2_	125.9, CH	51.5, CH_2_	122.6, CH
4	65.5, C	64.4, C	62.2, C	148.0, C
5	58.6, CH	51.3, CH	54.0, CH	38.9, C
6	138.2, C	135.1, C	134.5, C	32.0, CH_2_
7	148.8, CH	147.0, CH	152.6, CH	26.8, CH_2_
8	60.0, CH	56.6, CH	57.1, CH	38.8, C
9	45.6, CH	78.2, C	74.8, C	41.7, CH
10	17.3, CH_3_	63.4, CH_2_	22.5, CH_3_	41.8, CH
11	34.3, CH_2_	174.2, C	178.3, C	62.7, CH_2_
12	30.1, CH_2_	69.9, CH_2_	69.4, CH_2_	21.4, CH_3_
13	167.5, C	166.1, C	165.8, C	22.7, CH_3_
14	70.9, CH_2_	14.5, CH_3_	29.5, CH_3_	11.9, CH_3_
15	24.6, CH_3_	15.6, CH_3_	31.8, CH_3_	71.7, CH_2_
16	51.8, CH_3_	52.2, CH_3_	52.1, CH_3_	

**Table 3:** Growth inhibition (GI_50_, µM) of **1**–**9** against solid cancer cell lines.

Compounds	ACHN	MDA-MB-231	HCT-15	PC-3	NUGC-3	NCI-H23
**1**	>30	>30	>30	>30	>30	>30
**2**	>30	>30	>30	>30	>30	>30
**3**	>30	>30	>30	>30	>30	>30
**4**	2.44 ± 0.27	3.22 ± 0.15	3.12 ± 0.02	2.88 ± 0.06	2.23 ± 0.14	2.60 ± 0.02
**5**	2.15 ± 0.17	2.88 ± 0.39	2.46 ± 0.35	3.46 ± 0.03	2.37 ± 0.38	2.73 ± 0.35
**6**	2.31 ± 0.06	2.63 ± 0.15	2.35 ± 0.11	2.92 ± 0.20	2.41 ± 0.09	2.75 ± 0.11
**7**	>30	>30	>30	>30	>30	>30
**8**	1.97 ± 0.13	2.71 ± 0.10	3.23 ± 0.12	3.24 ± 0.58	1.97 ± 0.44	2.40 ± 0.12
**9**	>30	>30	>30	>30	>30	>30
Adr.	0.095 ± 0.005	0.073 ± 0.009	0.080 ± 0.007	0.084 ± 0.010	0.092 ± 0.023	0.070 ± 0.007

Adr.: Adriamycin as a positive control. GI_50_ value: The concentration that causes 50% growth inhibition, a smaller value stands for the more potent inhibition.

**Table 4:** Growth inhibition (GI_50_, µM) of **1**–**9** against blood cancer cell lines.

Compounds	HL-60	Raji	K562	RPMI-8402	NALM6	U266	WSU-DLCL2
**1**	>30	>30	>30	>30	>30	>30	>30
**2**	>30	>30	>30	>30	>30	>30	>30
**3**	16.39 ± 2.38	>30	>30	>30	>30	>30	>30
**4**	>30	>30	28.23 ± 0.85	26.39 ± 0.63	17.75 ± 1.73	>30	>30
**5**	>30	>30	>30	>30	>30	>30	>30
**6**	22.58 ± 0.74	21.30 ± 3.88	8.37 ± 0.26	8.85 ± 0.43	10.65 ± 0.74	>30	14.03 ± 1.17
**7**	>30	>30	>30	>30	>30	>30	>30
**8**	>30	>30	17.94 ± 1.05	25.37 ± 2.90	24.77 ± 1.22	>30	28.26 ± 3.45
**9**	>30	>30	>30	>30	>30	>30	>30
Adr.	0.017 ± 0.001	0.008 ± 0.001	0.090 ± 0.002	0.017 ± 0.000	0.003 ± 0.000	0.035 ± 0.001	0.004 ± 0.000

Adr.: Adriamycin as a positive control.

## Data Availability

The data presented in the article are available in the Supplementary Materials.

## Supplementary Materials

The following supporting information can be downloaded at: https://www.mdpi.com/article/10.3390/md21060361/s1, Figures S1–S34: 1D and 2D NMR, and ECD experimental spectra and HRESIMS data of **1**, **4**, **7**, and **9**. Figures S35 and S36: Results of the cytotoxicity test of **1**–**9** against six solid and seven blood cancer cell lines.
