# Supplementary material for: Sesquiterpenes from Streptomyces qinglanensis and Their Cytotoxic Activity
Source: Mar Drugs. 2023 Jun 16;21(6):361. doi: 10.3390/md21060361 (PMC10305546; doi:10.3390/md21060361)
Supplement: Supplementary file 1 [file marinedrugs-21-00361-s001.zip › marinedrugs-2448841-supplementary.pdf]

Sesquiterpenes from *Streptomyces qinglanensis* and Their Cytotoxic Activity

Figure S1. ^{1}H NMR spectrum of **1**. 3
Figure S2. ^{13}C NMR spectrum of **1**. 4
Figure S3. HSQC spectrum of **1**. 5
Figure S4. ^{1}H-^{1}H COSY spectrum of **1**. 6
Figure S5. HMBC spectrum of **1**. 7
Figure S6. NOESY spectrum of **1**. 8
Figure S7. HRESI MS data of **1**. 9
Figure S8. Comparison of chemical shifts of **4** and **6**. 10
Figure S9. ^{1}H NMR spectrum of **4**. 11
Figure S10. ^{13}C NMR spectrum of **4**. 12
Figure S11. HSQC spectrum of **4**. 13
Figure S12. ^{1}H-^{1}H COSY spectrum of **4**. 14
Figure S13. HMBC spectrum of **4**. 15
Figure S14. 1D selective NOESY spectrum of **4** (irradiated at H-8). 16
Figure S15. 1D selective NOESY spectrum of **4** (irradiated at H-5). 17
Figure S16. HRESIMS data of **4**. 18
Figure S17. ^{1}H NMR spectrum of **7**. 19
Figure S18. ^{13}C NMR spectrum of **7**. 20
Figure S19. HSQC spectrum of **7**. 21
Figure S 20. ^{1}H-^{1}H COSY spectrum of **7**. 22
Figure S21. HMBC spectrum of **7**. 23
Figure S22. NOESY spectrum of **7**. 24
Figure S23. 1D selective NOESY spectrum of **7** (irradiated at H-5). 25
Figure S24. HRESIMS data of **7**. 26
Figure S25. Comparison NMR data of bolinane A (**9**) with bolinaquinone. 27
Figure S26. ^{1}H NMR spectrum of **9**. 28

Figure S27. ^{13}C NMR spectrum of **9**. 29
Figure S28. HSQC spectrum of **9**. 30
Figure S29. ^{1}H-^{1}H COSY spectrum of **9**. 31
Figure S30. HMBC spectrum of **9**. 32
Figure S31. Selective 1D NOESY spectrum of **9** (irradiated at H_3-12). 33
Figure S32. Selective 1D NOESY spectrum of **9** (irradiated at H_3-14). 34
Figure S33. Selective 1D NOESY spectrum of **9** (irradiated at H_3-13). 35
Figure S34. HRESIMS data of **9**. 36
Figure S35. Results of the cytotoxicity test of **1-9** against six solid cancer cell lines. 39
Figure S36. Results of the cytotoxicity test of **1-9** against seven blood cancer cell lines. 43

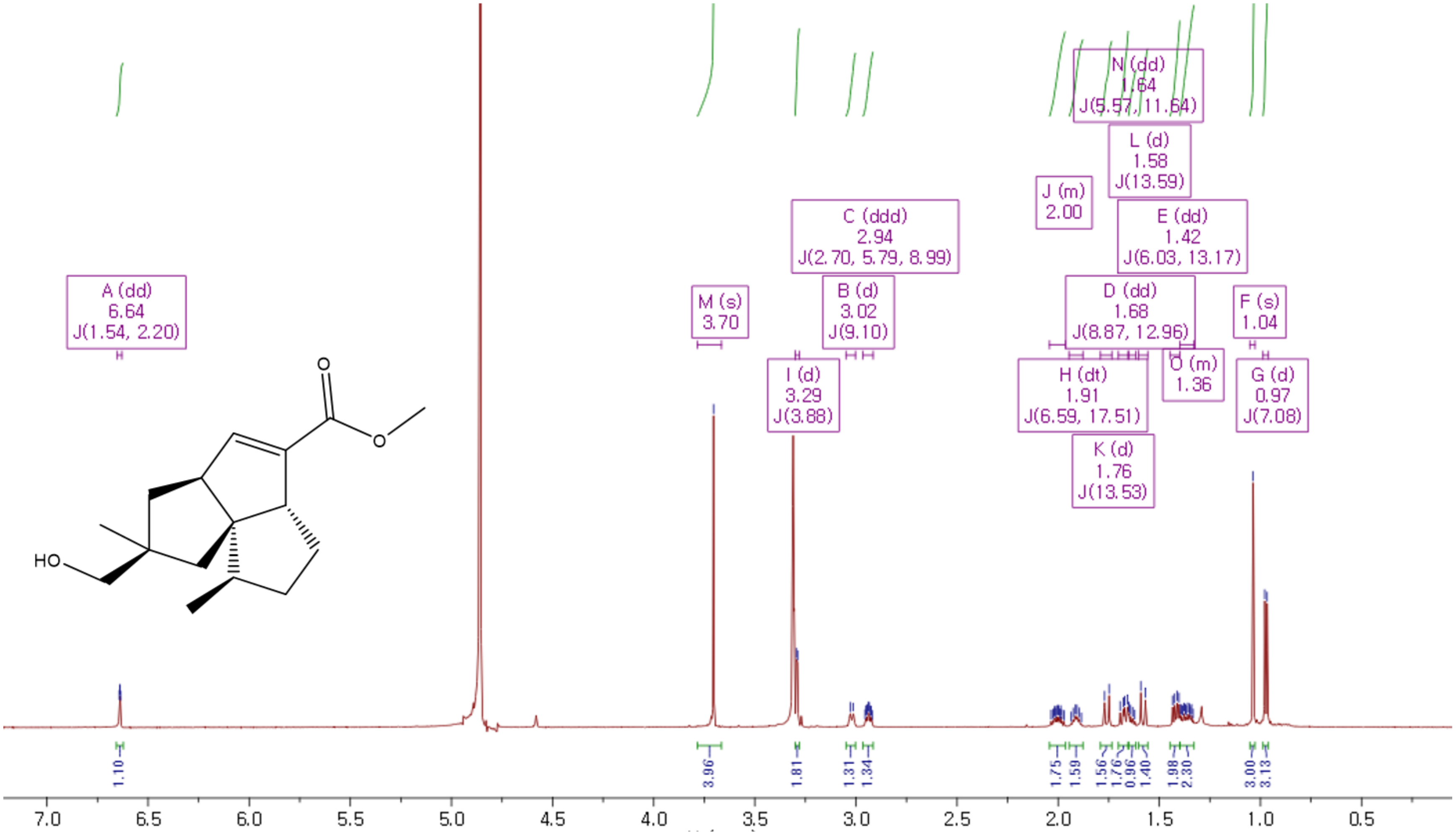

Figure S1. ^{1}H NMR spectrum of **1**.

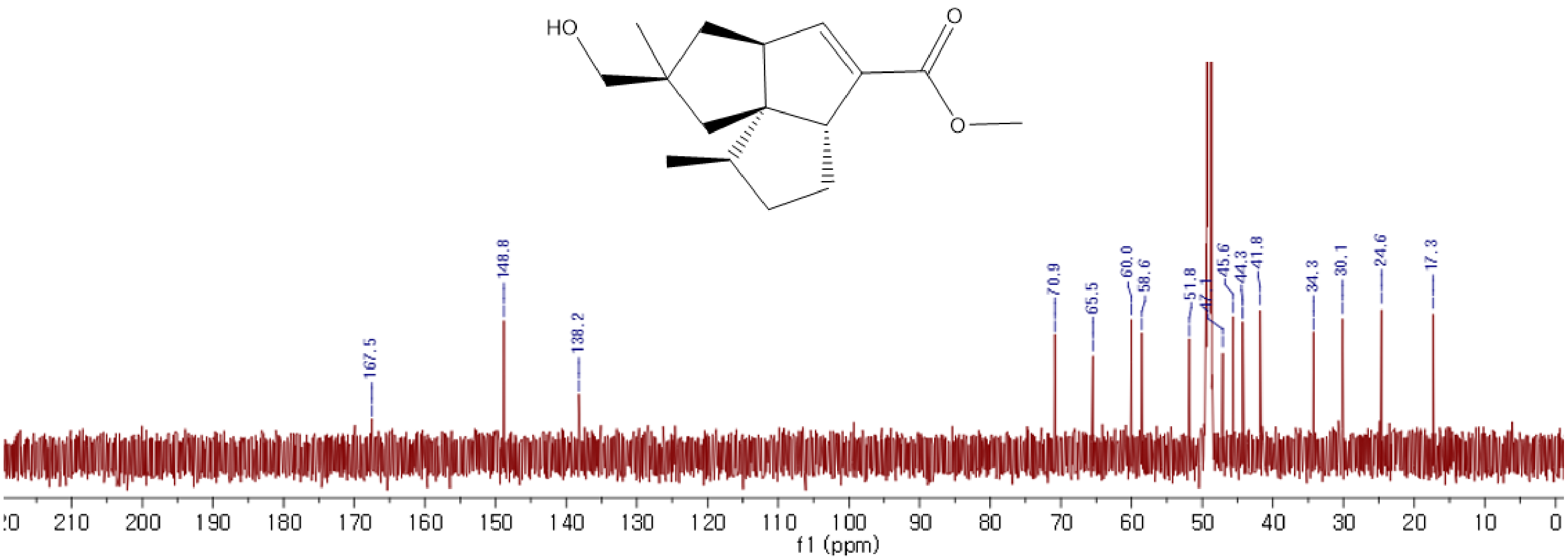

Figure S2. ^{13}C NMR spectrum of **1**.

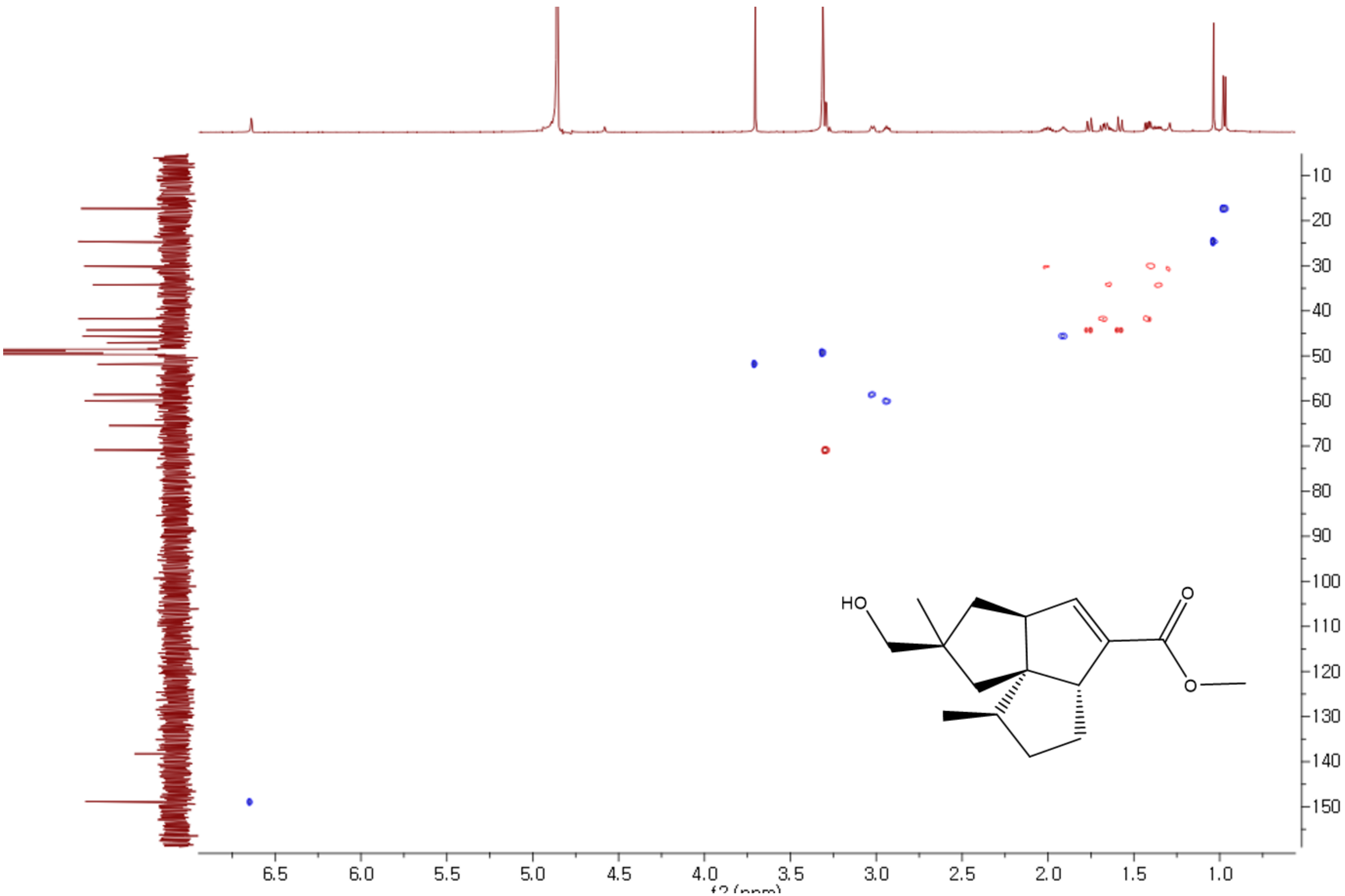

Figure S3. HSQC spectrum of **1**.

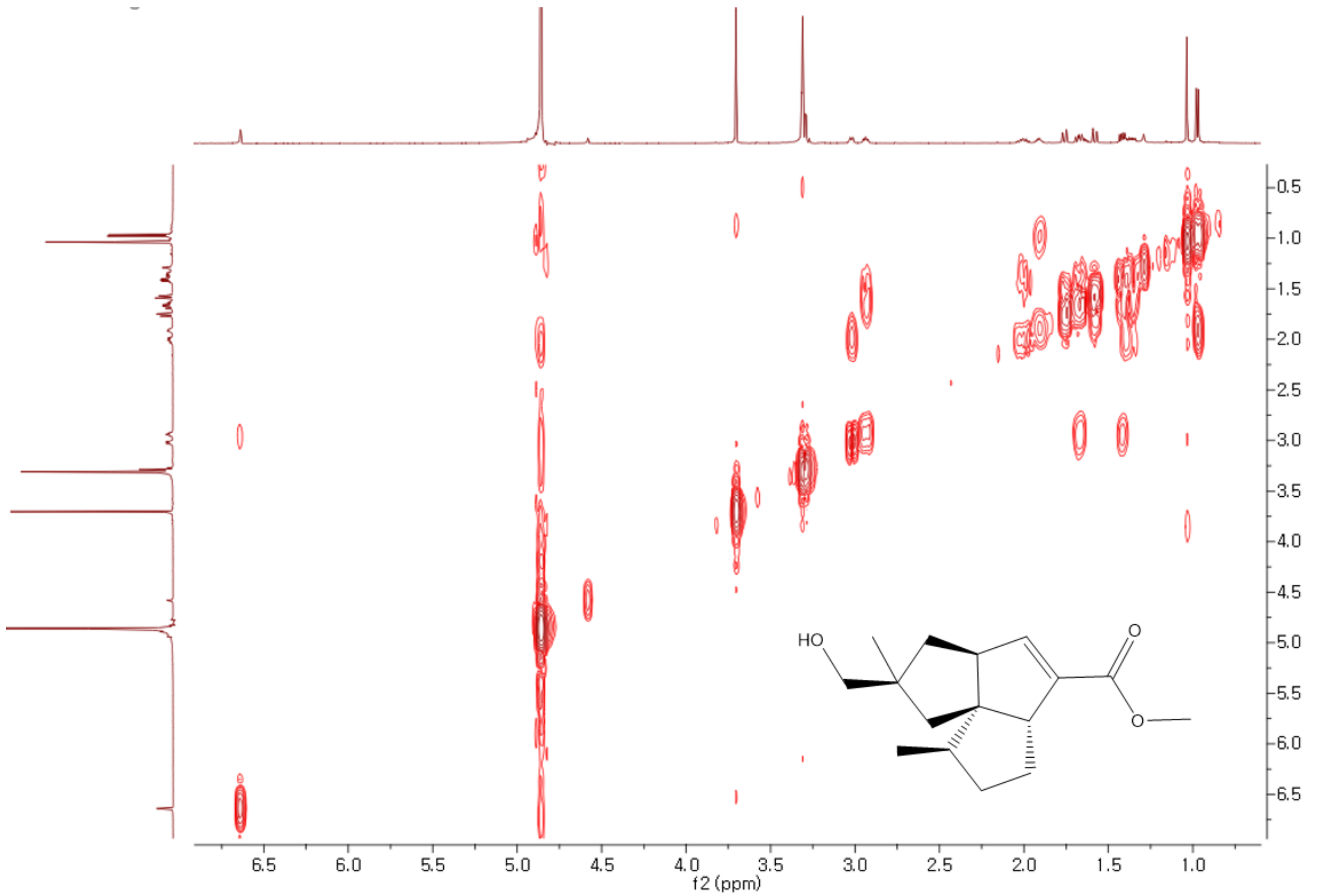

Figure S4. ^{1}H-^{1}H COSY spectrum of **1**.

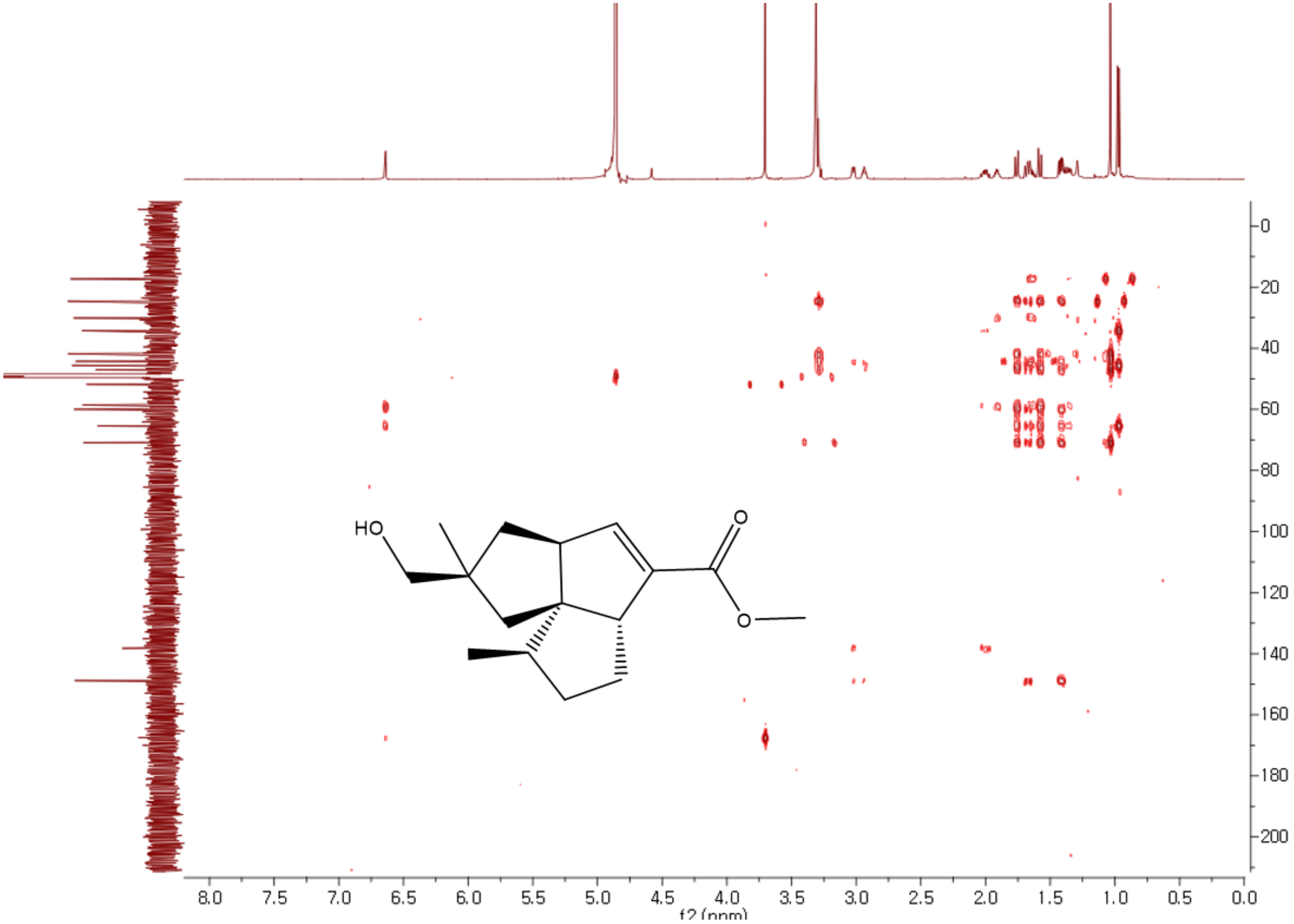

Figure S5. HMBC spectrum of **1**.

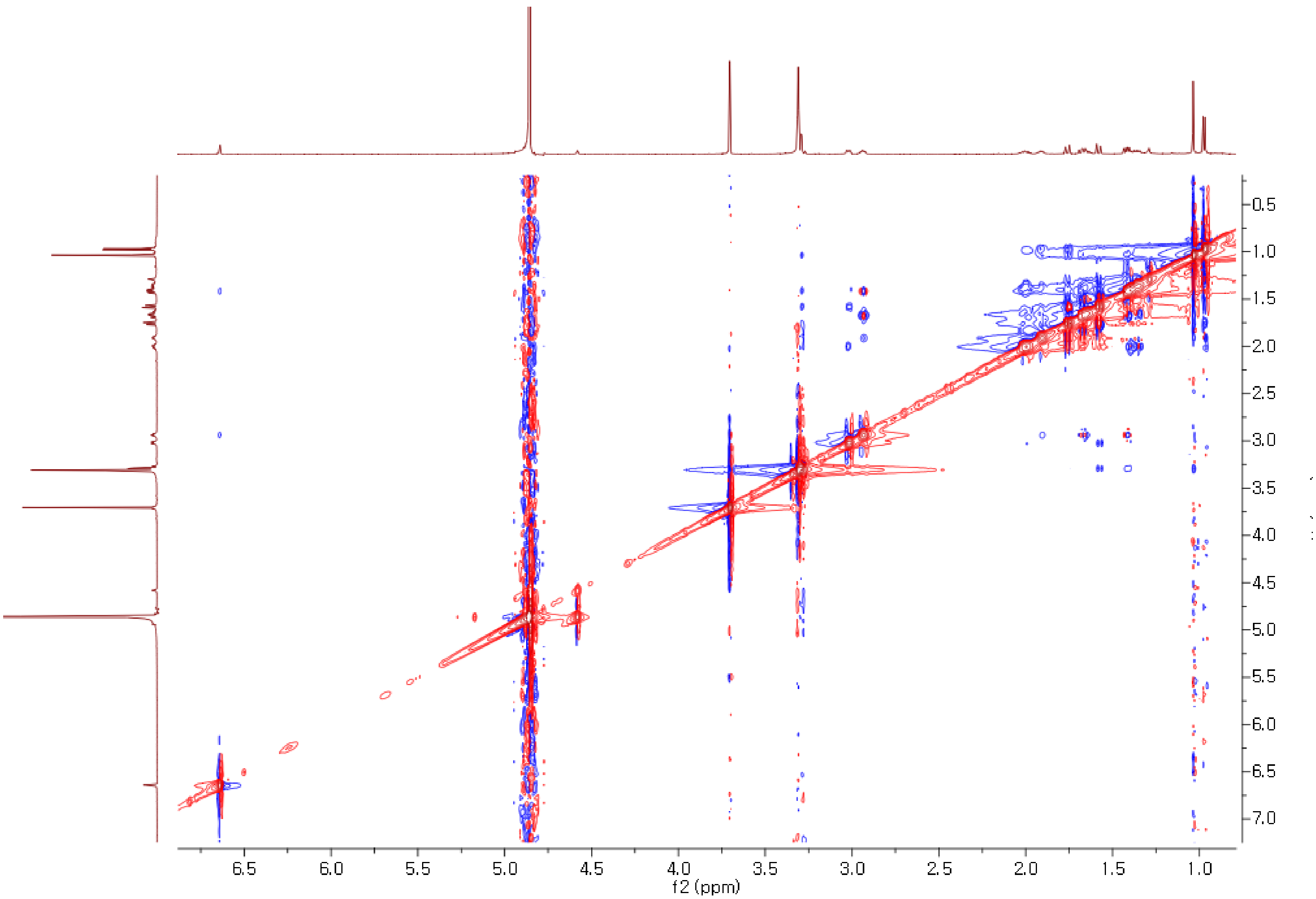

Figure S6. NOESY spectrum of **1**.

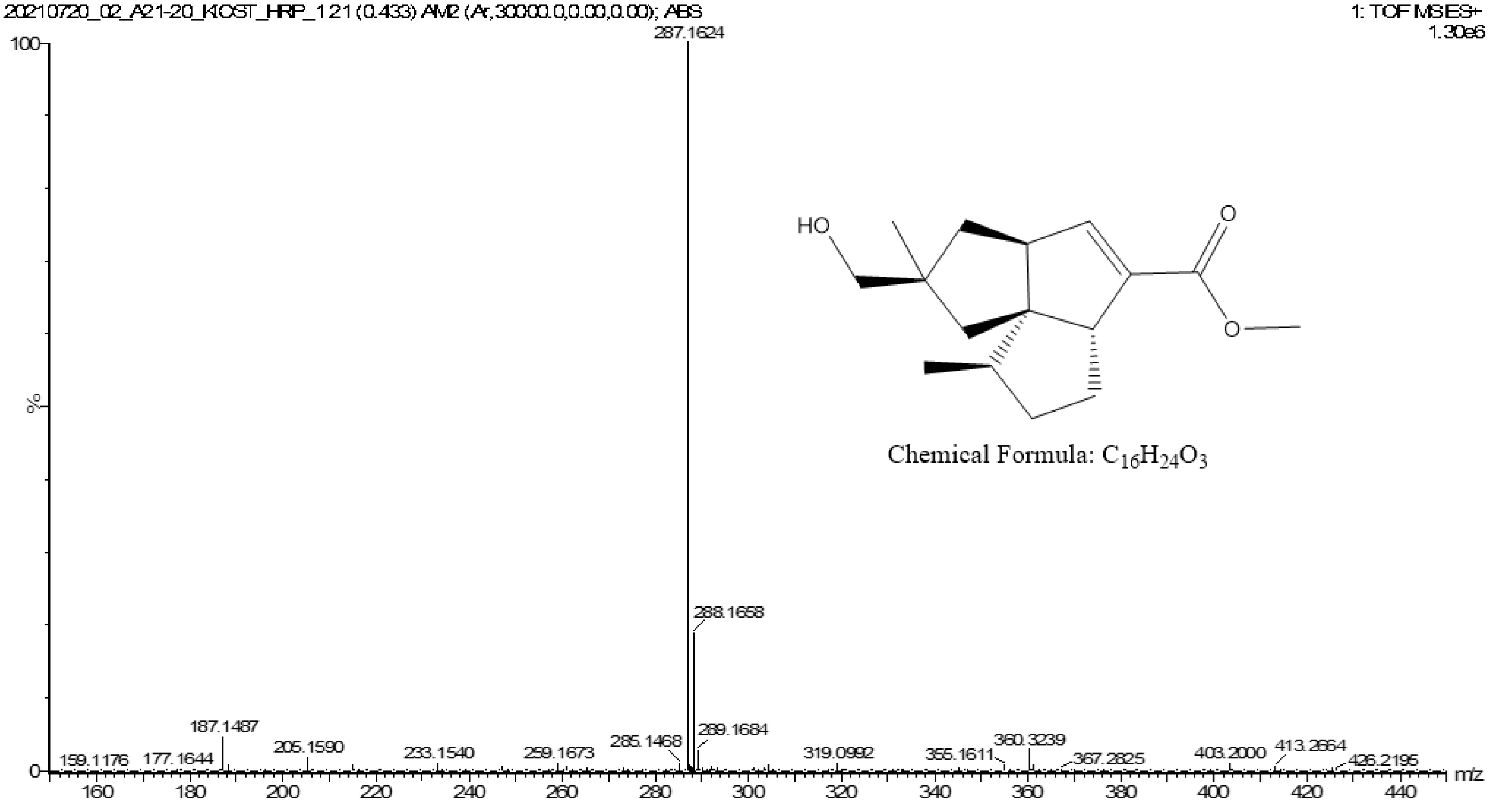

Mass	Calc. Mass	mDa	PPM	DBE	i-FIT	Norm	Conf(%)	Formula
287.1624	287.1623	0.1	0.3	4.5	1355.7	0.000	100.00	C16 H24 O3 Na

Figure S7. HRESI MS data of **1**.

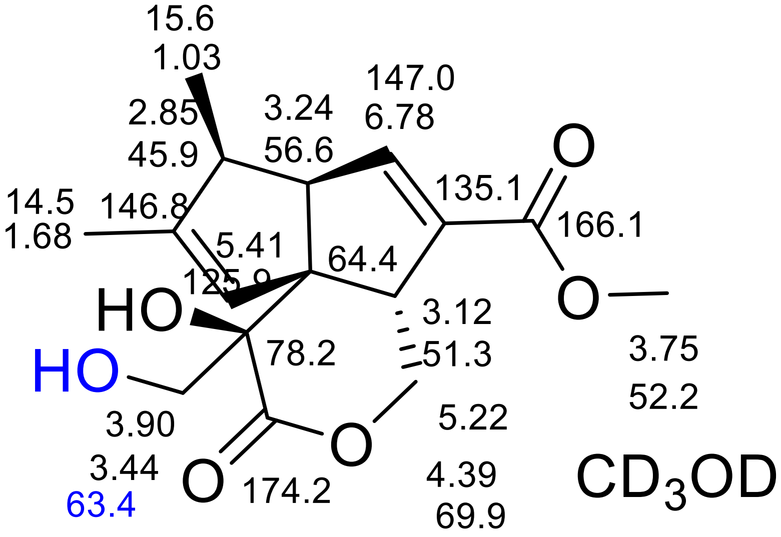

4

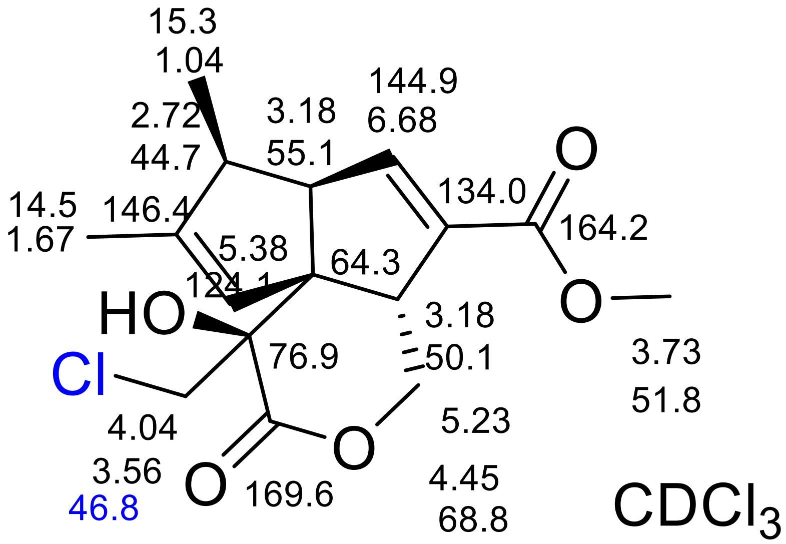

AA-57 methyl ester (**6**)

J Antibiot (Tokyo). **1978** Jul; *31* (7) : 729-31.
doi: 10.7164/antibiotics.31.729.

Figure S8. Comparison of chemical shifts of **4** and **6**.

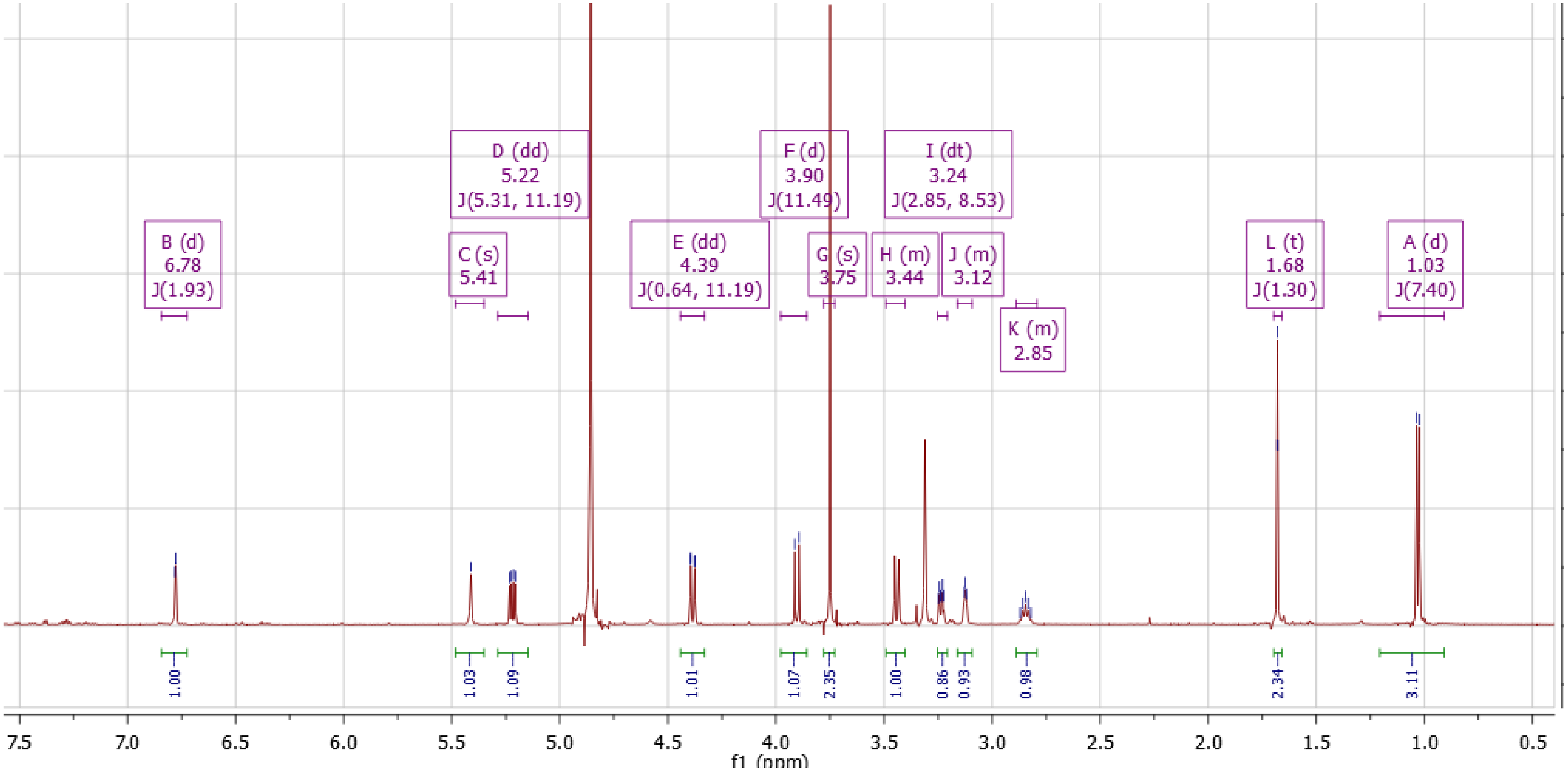

Figure S9. ^{1}H NMR spectrum of **4**.

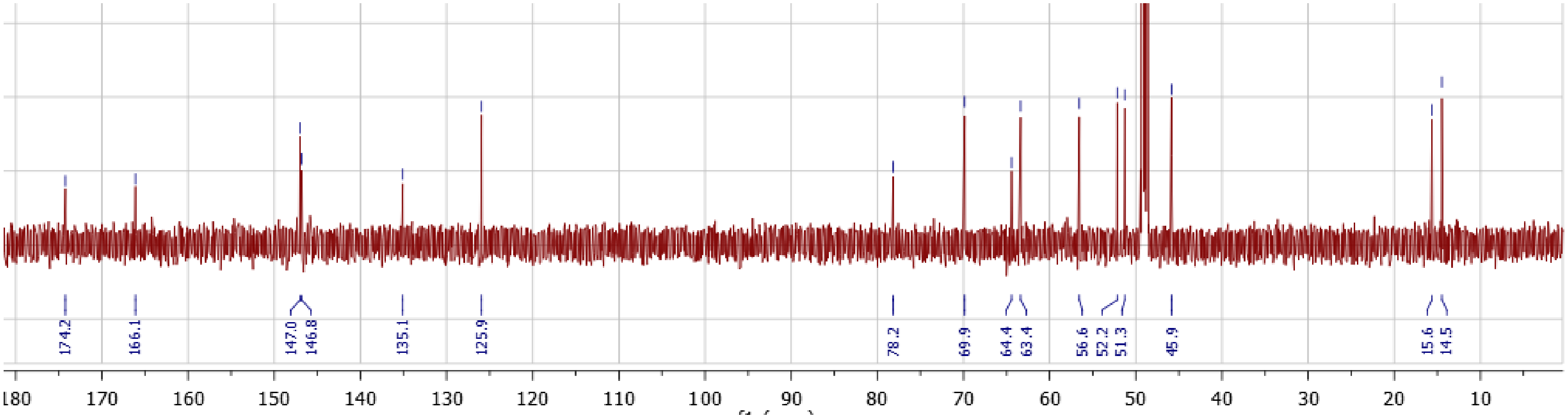

Figure S10. ^{13}C NMR spectrum of **4**.

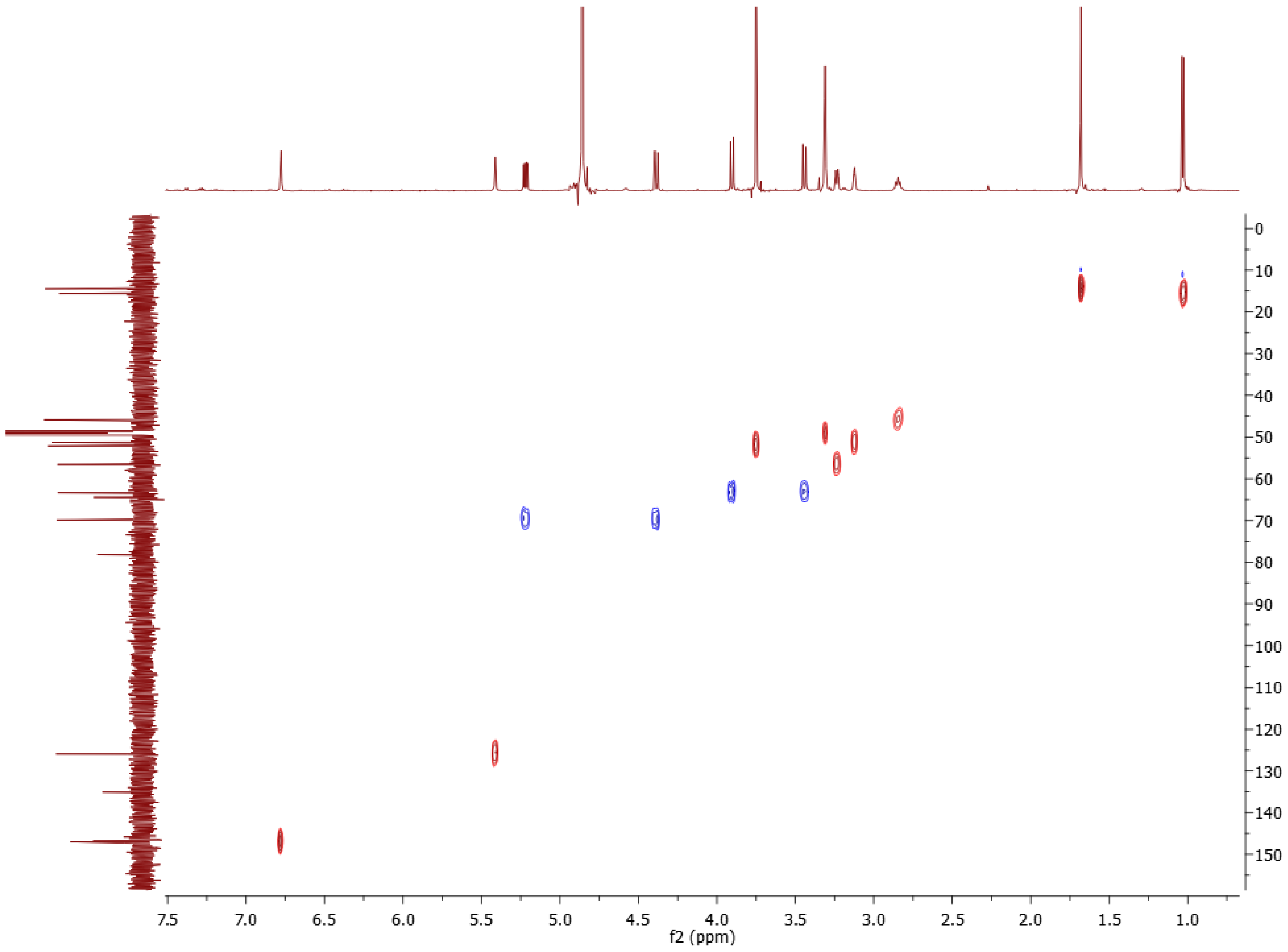

Figure S11. HSQC spectrum of **4**.

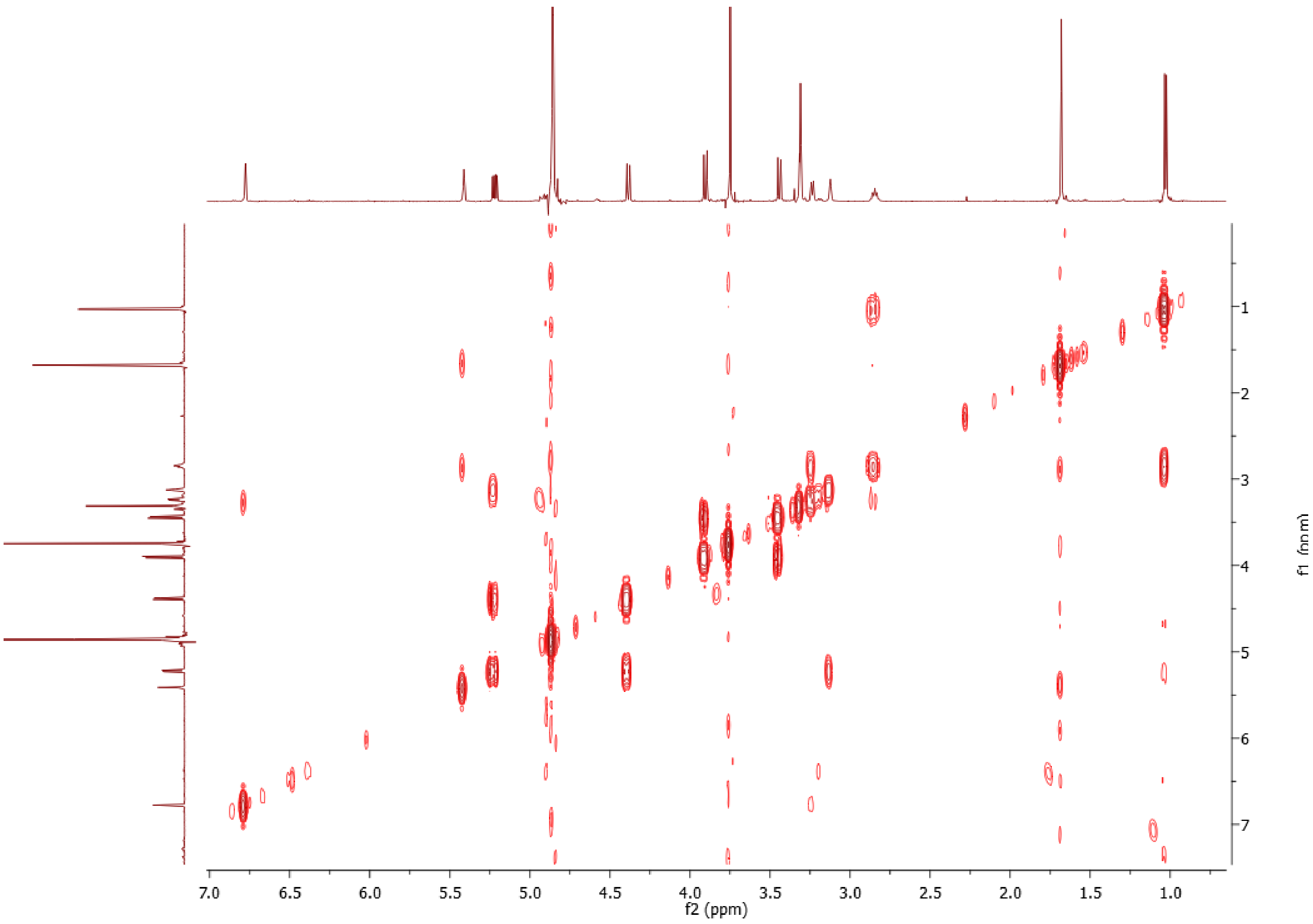

Figure S12. ^{1}H-^{1}H COSY spectrum of **4**.

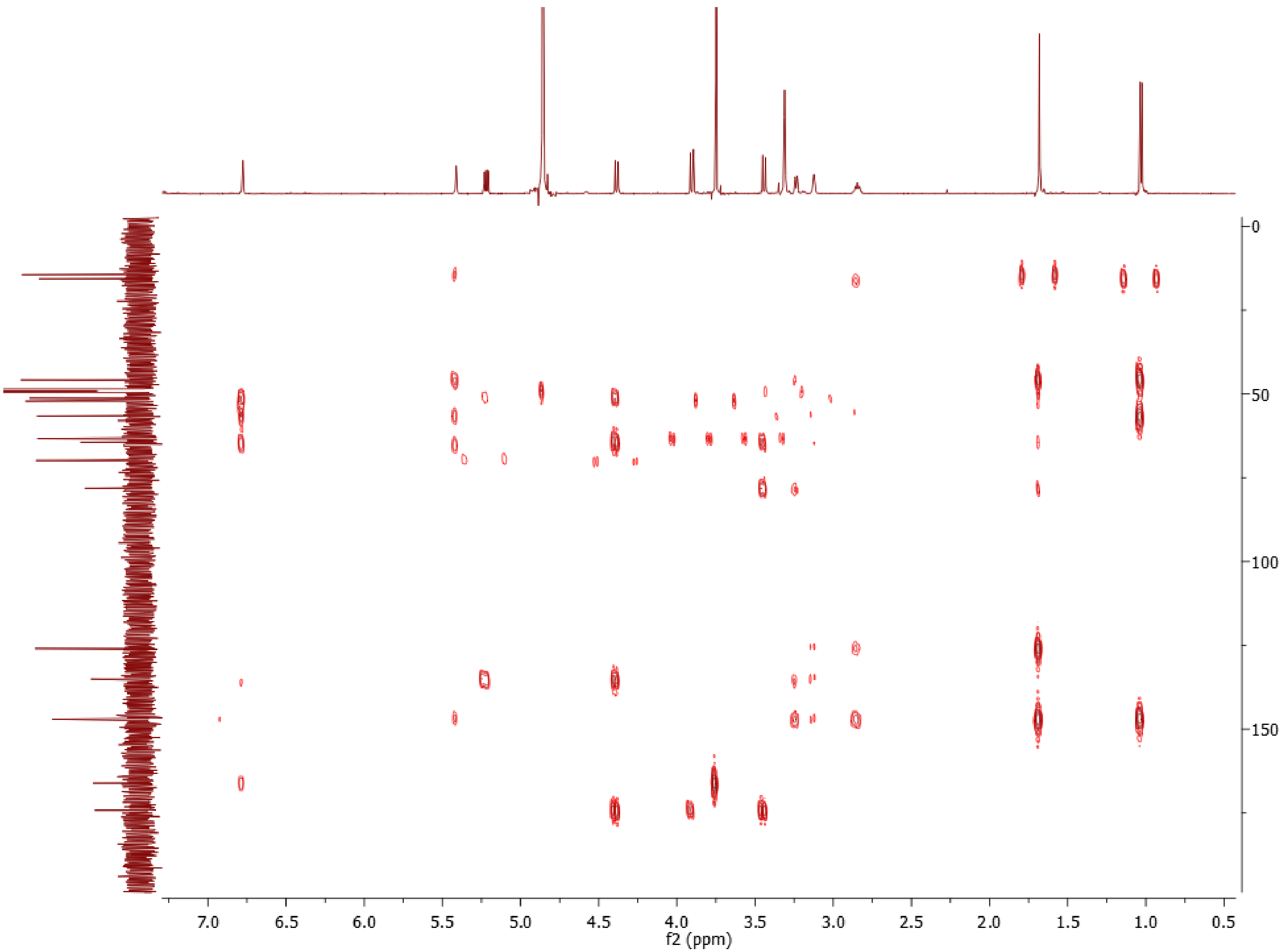

Figure S13. HMBC spectrum of **4**.

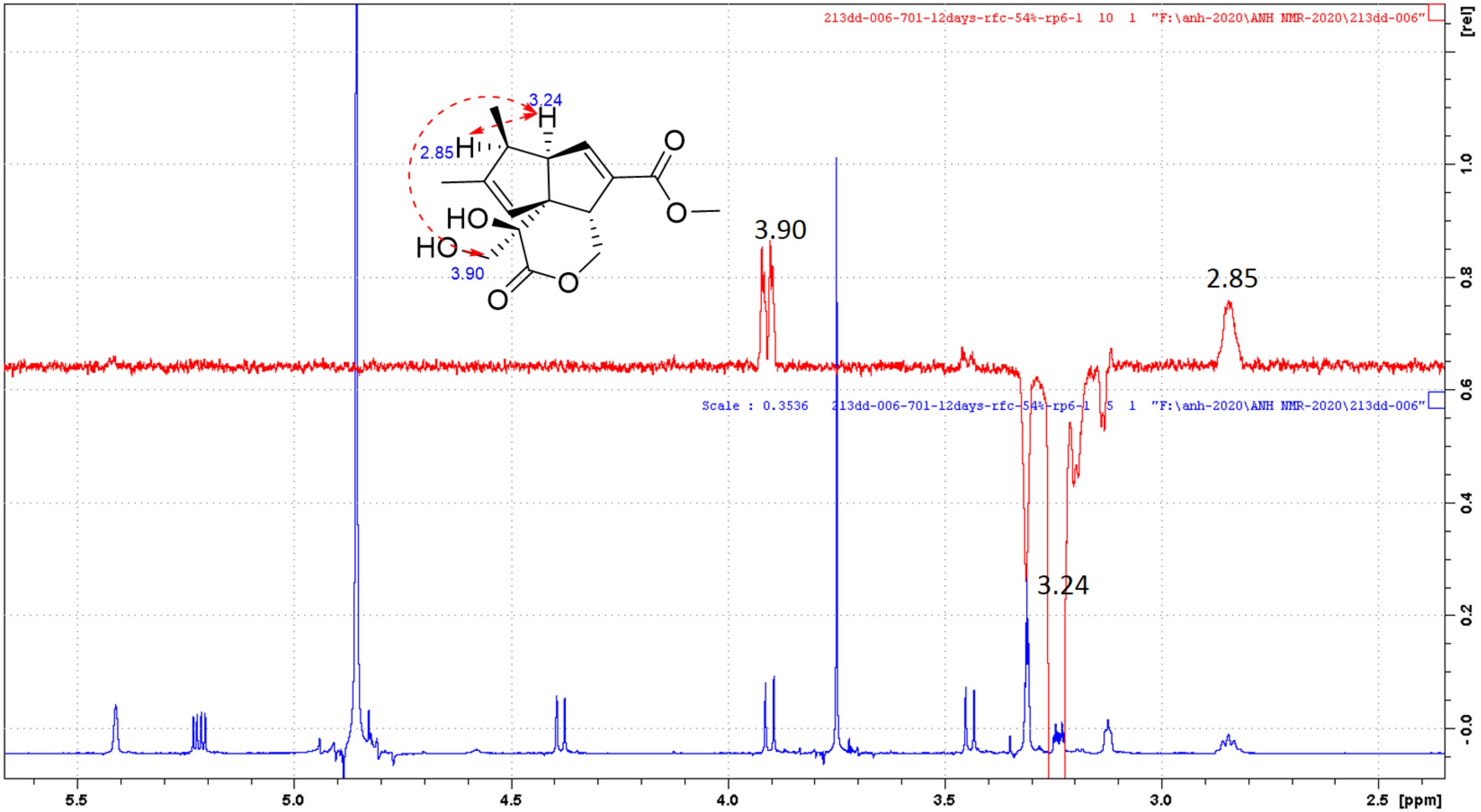

Figure S14. 1D selective NOESY spectrum of **4** (irradiated at H-8).

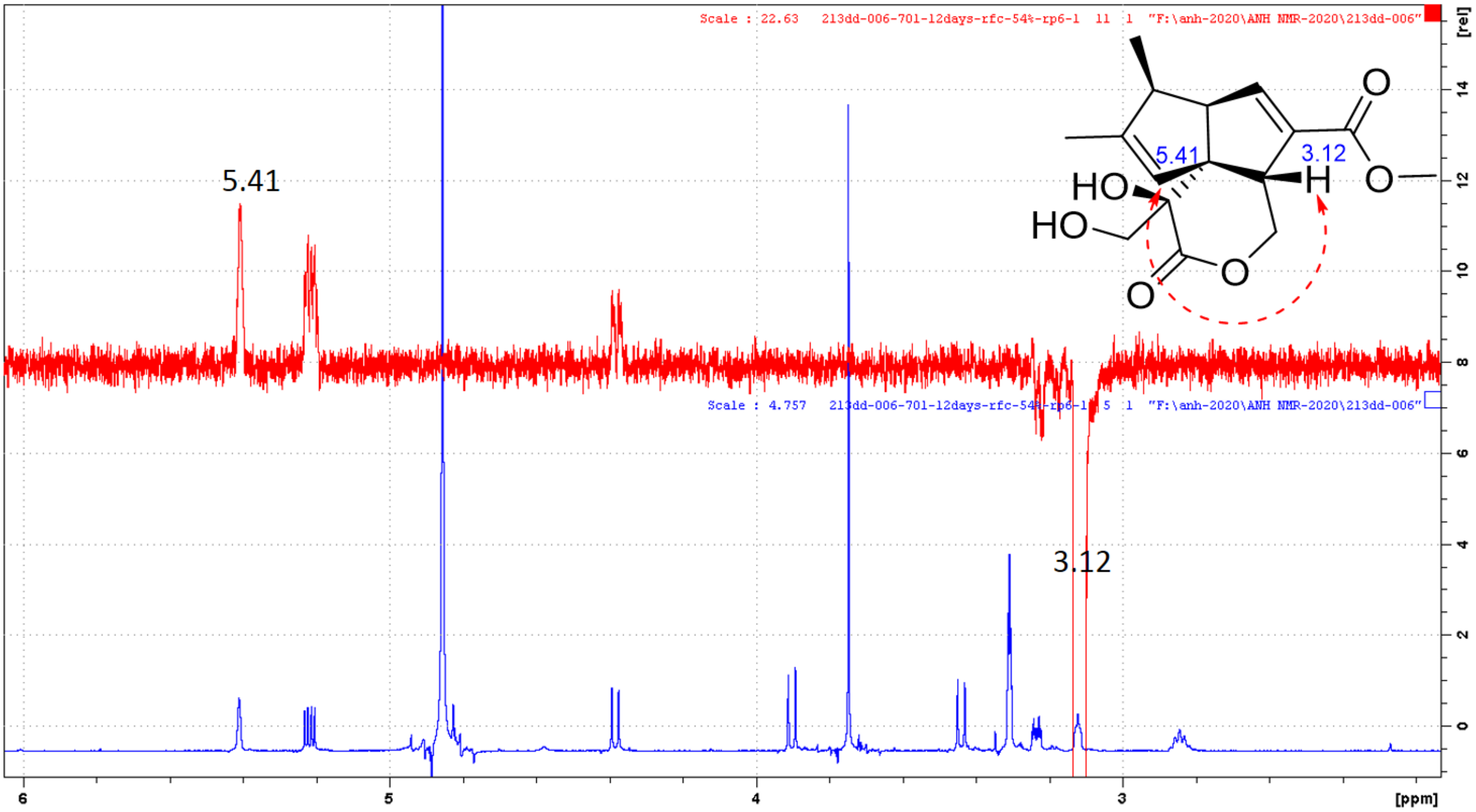

Figure S15. 1D selective NOESY spectrum of **4** (irradiated at H-5).

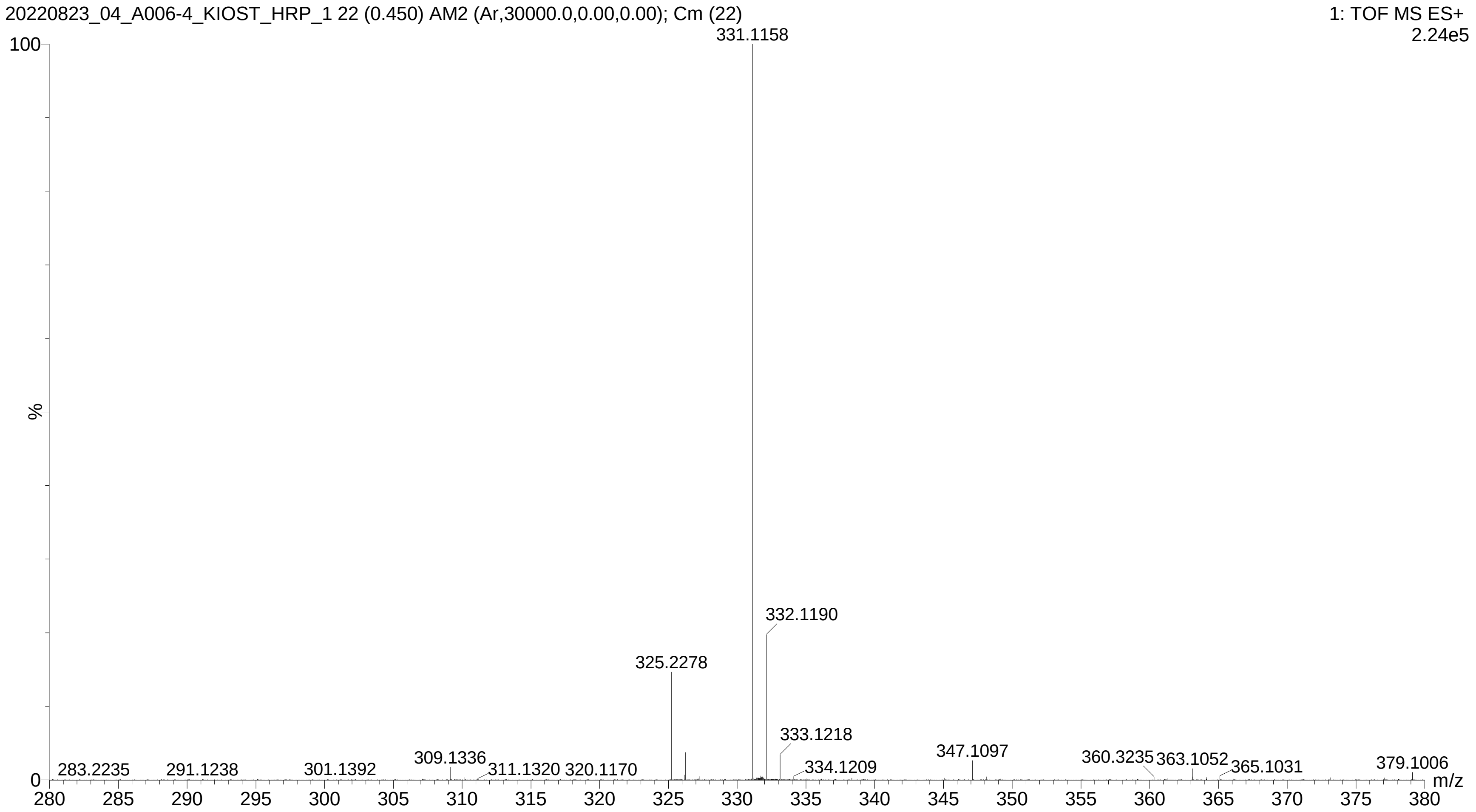

Mass	Calc. Mass	mDa	PPM	DBE	i-FIT	Norm	Conf(%)	Formula
331.1158	331.1158	0.0	0.0	6.5	845.9	n/a	n/a	C16 H20 O6 Na

Figure S16. HRESIMS data of **4**.

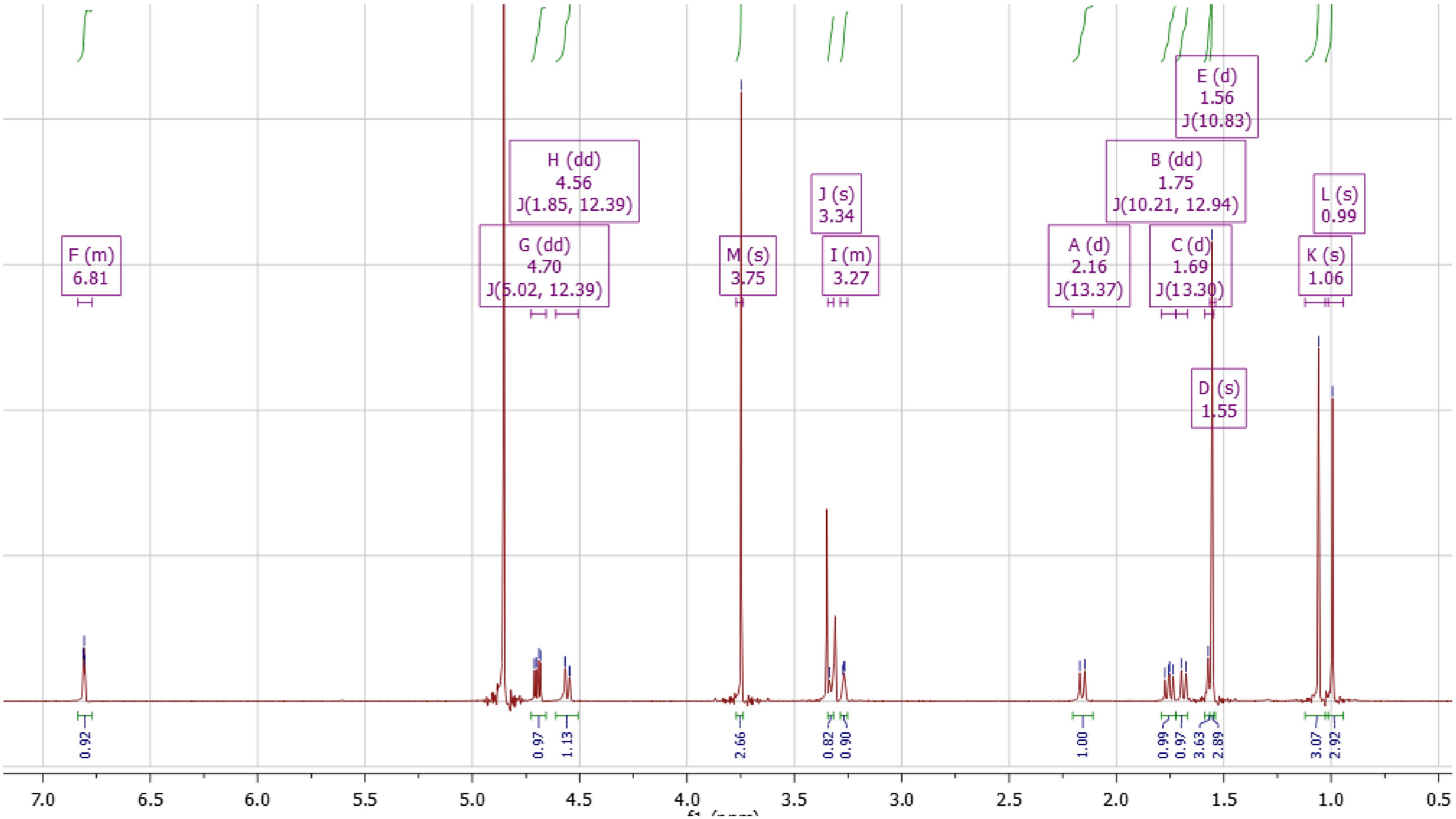

Figure S17. ^{1}H NMR spectrum of **7**.

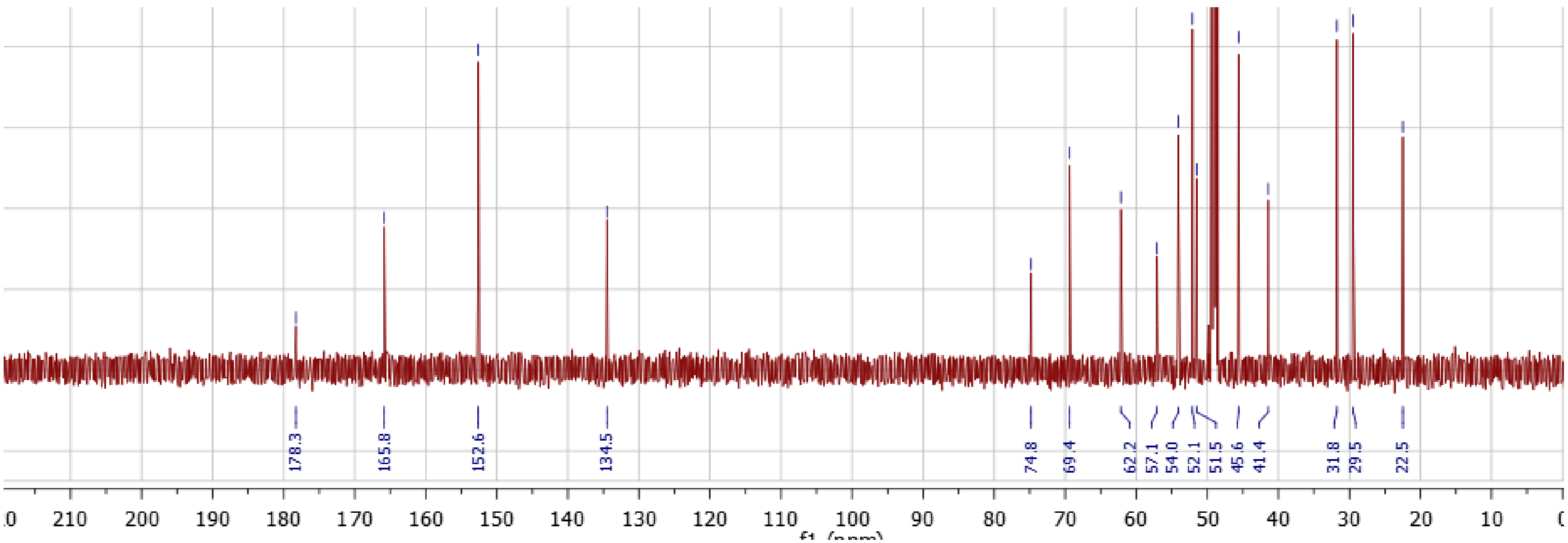

Figure S18. ^{13}C NMR spectrum of **7**.

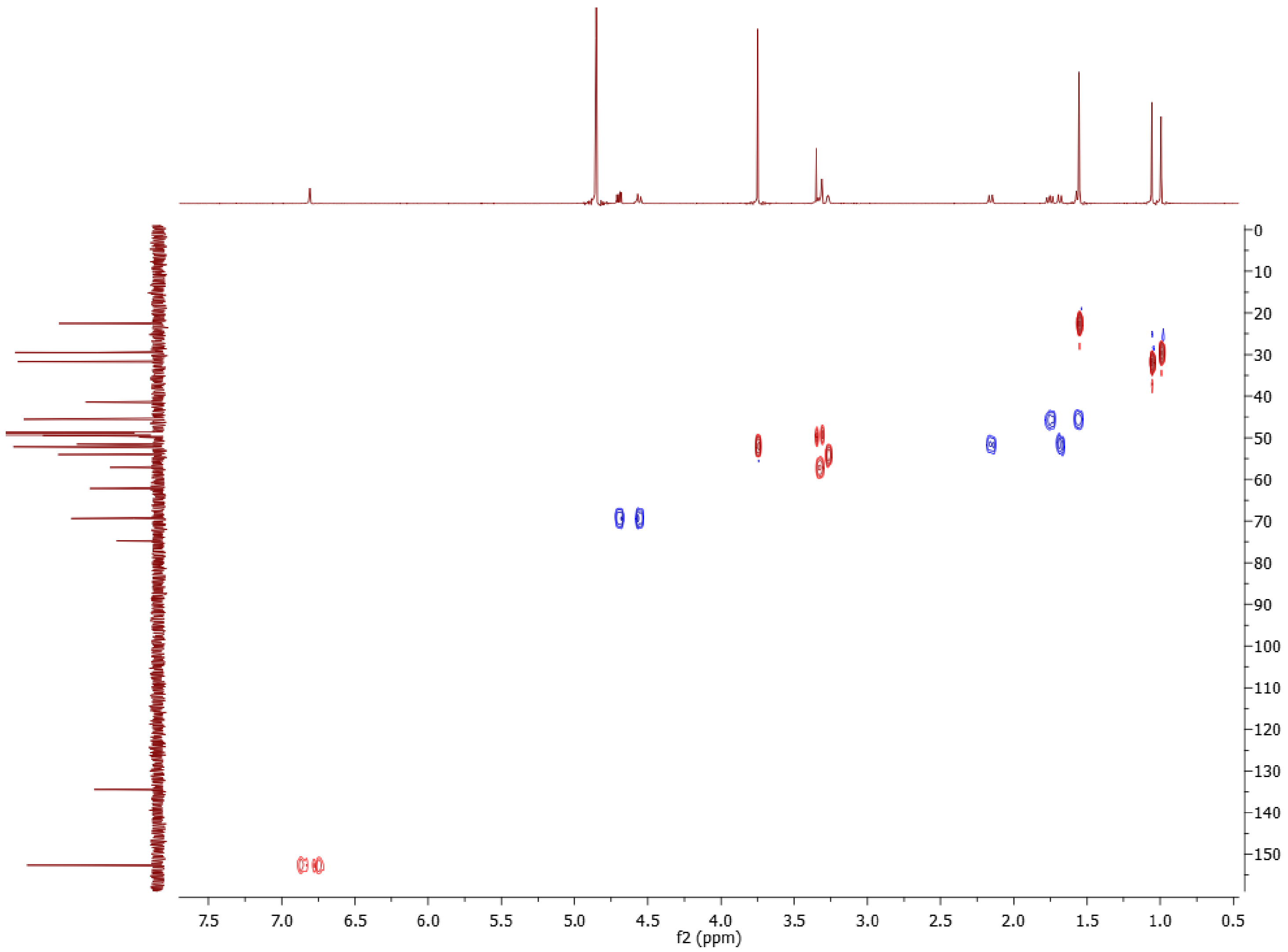

Figure S19. HSQC spectrum of **7**.

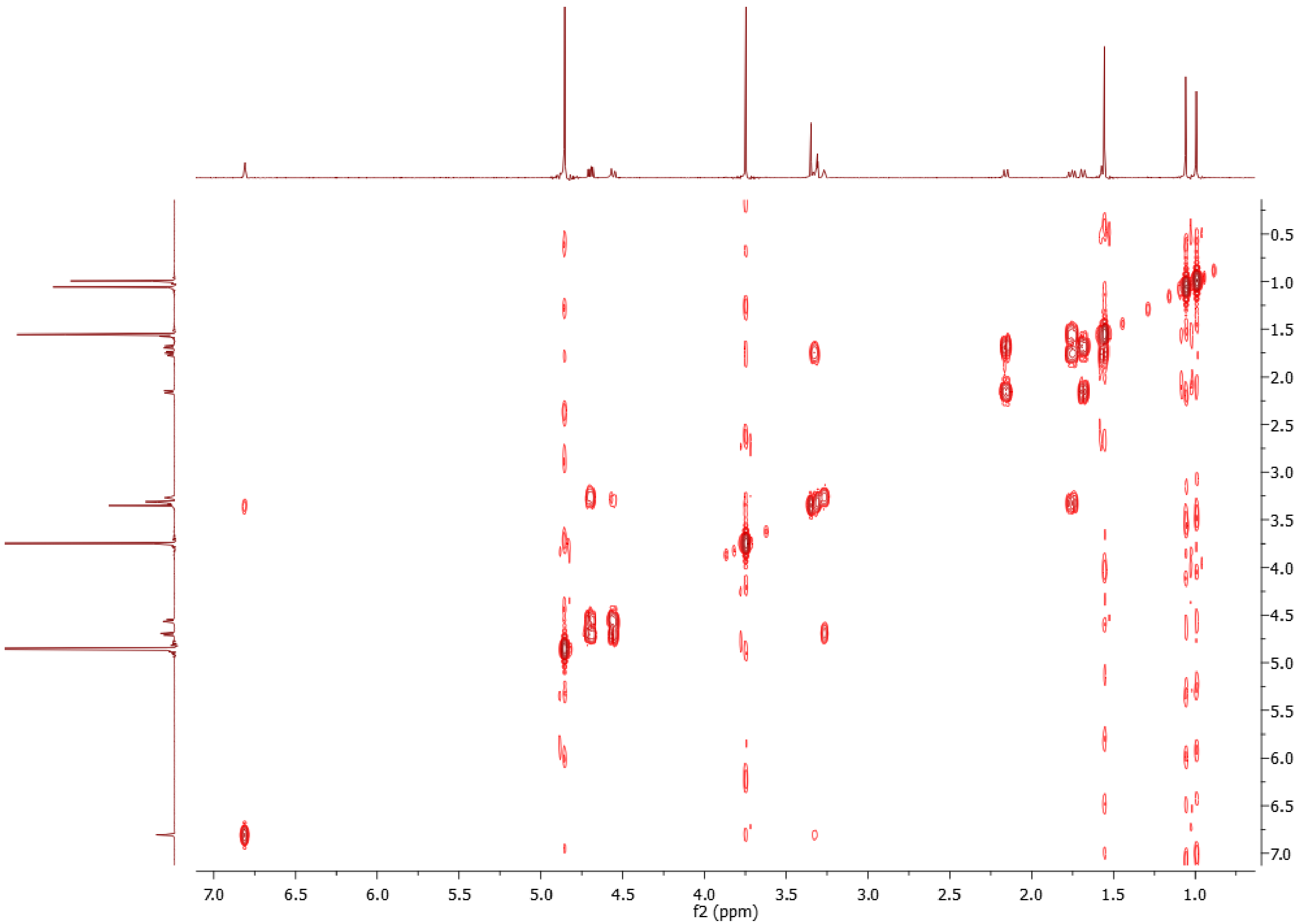

Figure S 20. ^{1}H-^{1}H COSY spectrum of **7**.

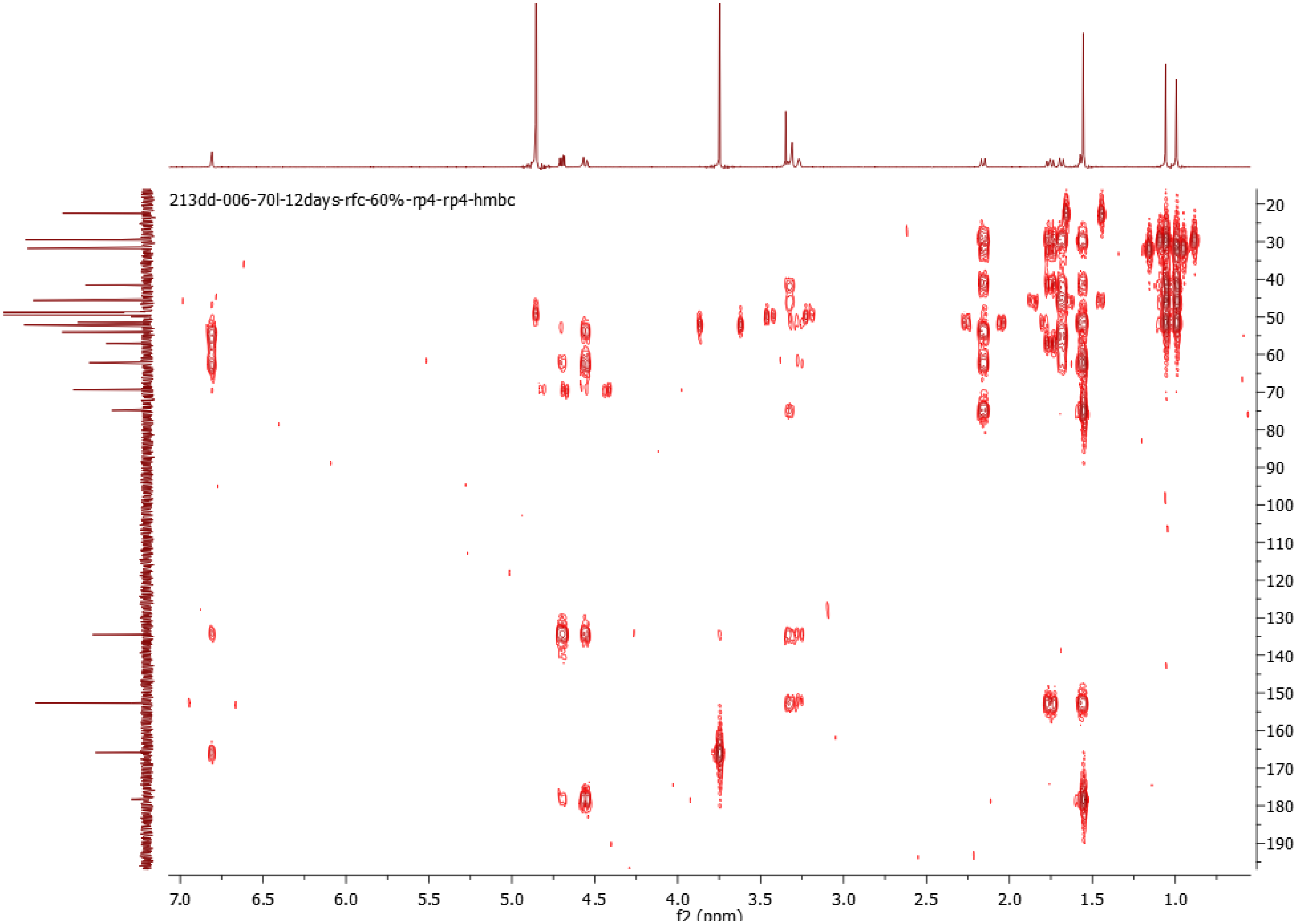

Figure S21. HMBC spectrum of **7**.

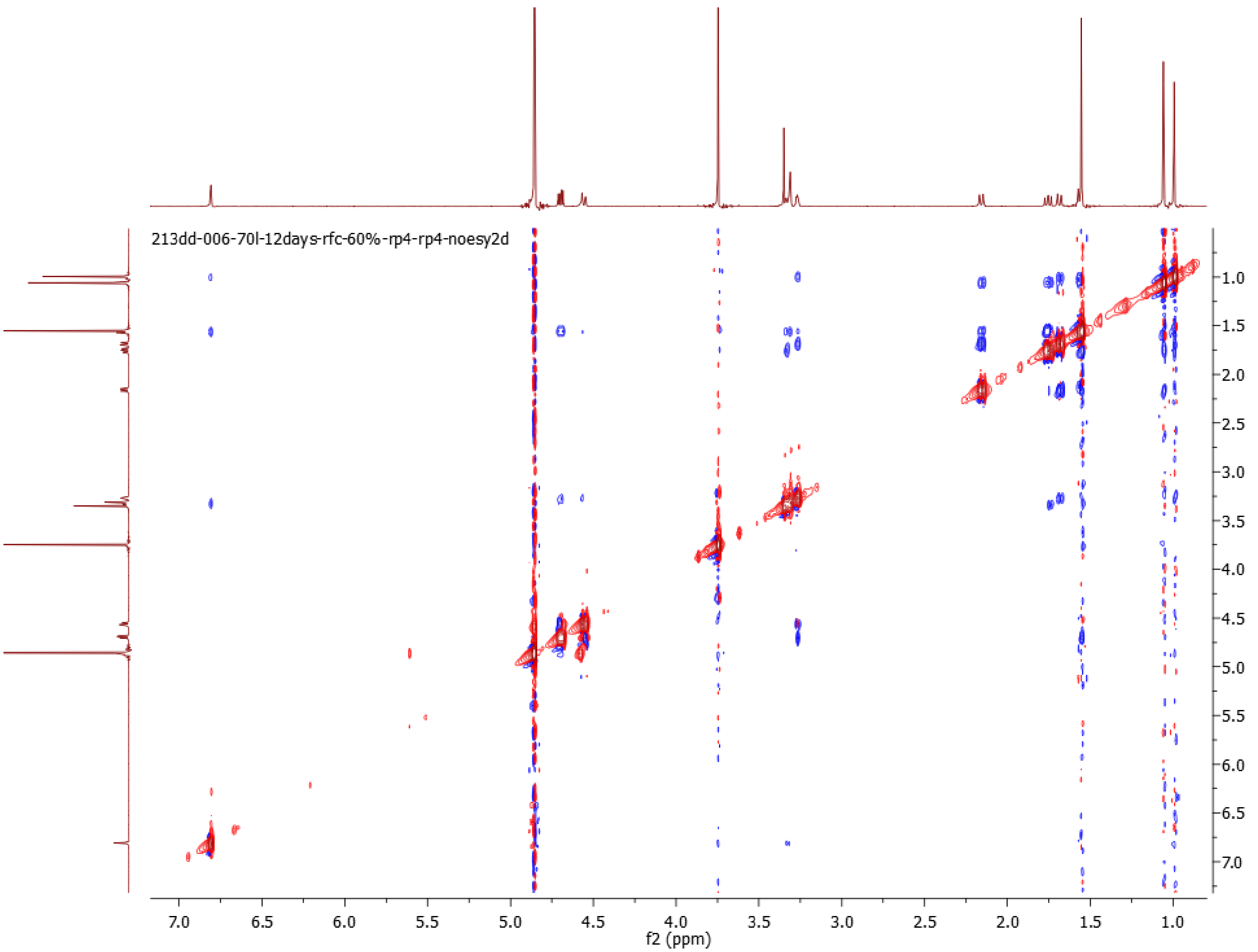

Figure S22. NOESY spectrum of **7**.

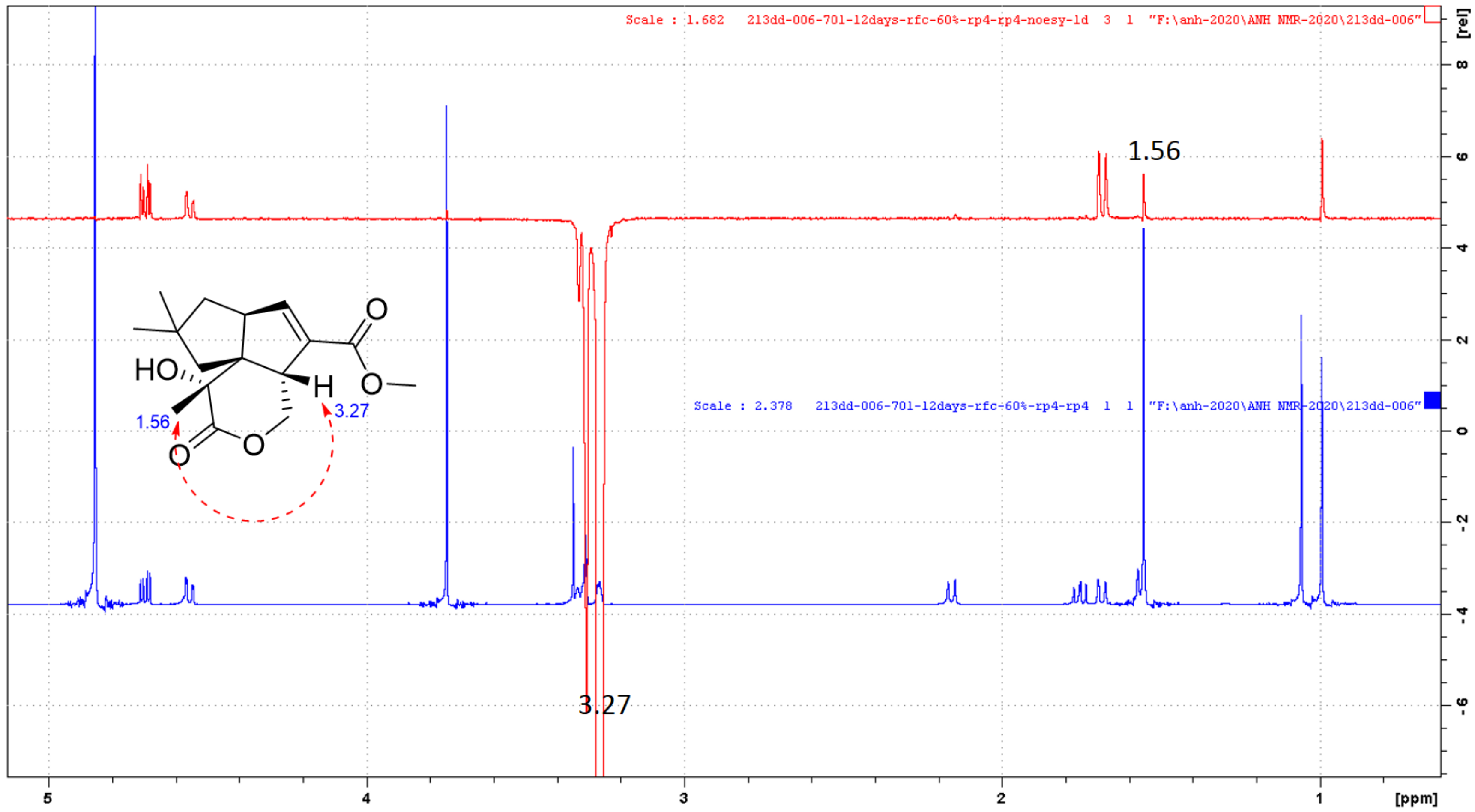

Figure S23. 1D selective NOESY spectrum of **7** (irradiated at H-5).

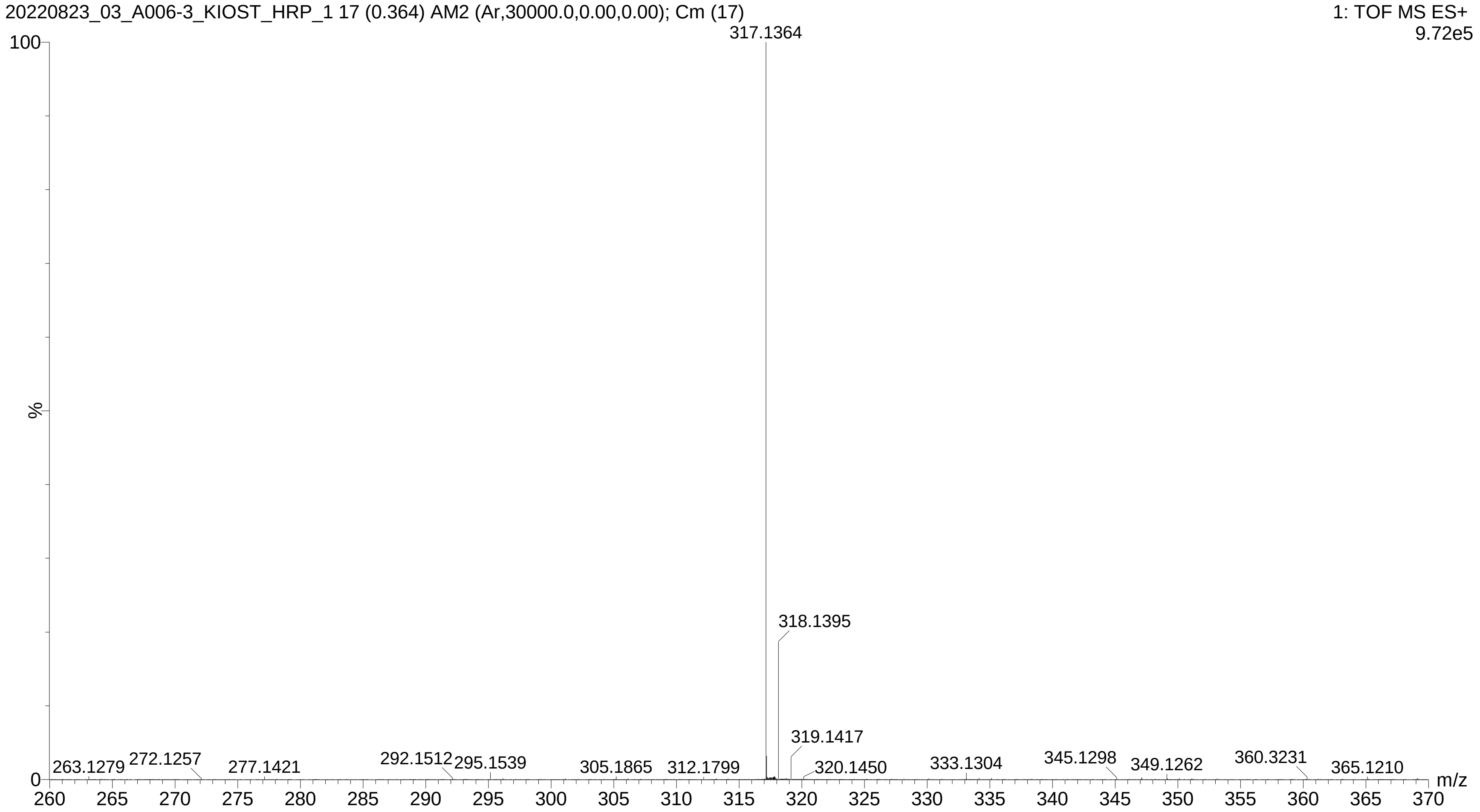

Mass	Calc. Mass	mDa	PPM	DBE	i-FIT	Norm	Conf(%)	Formula
317.1364	317.1365	-0.1	-0.3	5.5	1108.5	n/a	n/a	C16 H22 O5 Na

Figure S24. HRESIMS data of **7**.

9

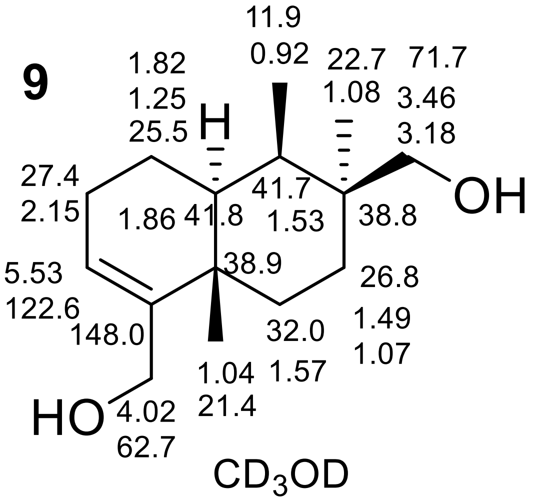

CD_3OD

Bolinaquinone

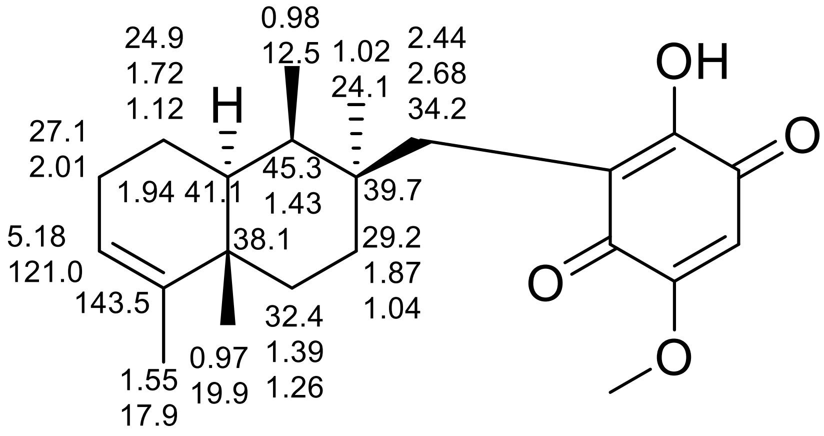

C_5D_5N

J. Org. Chem. **1998**, *63*, 8042-8044,
10.1021/jo981037t

Figure S25. Comparison NMR data of bolinane A (**9**) with bolinaquinone.

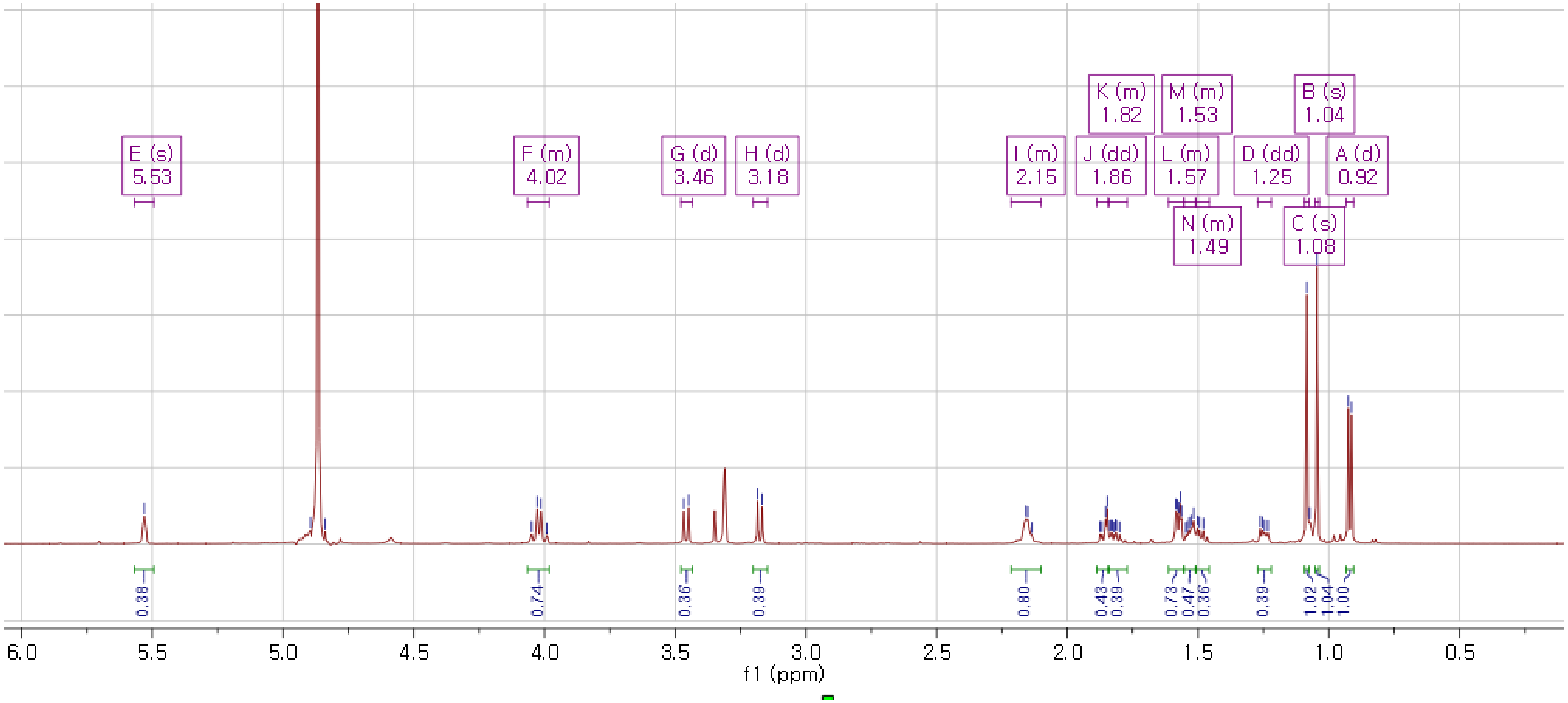

Figure S26. ^{1}H NMR spectrum of **9**.

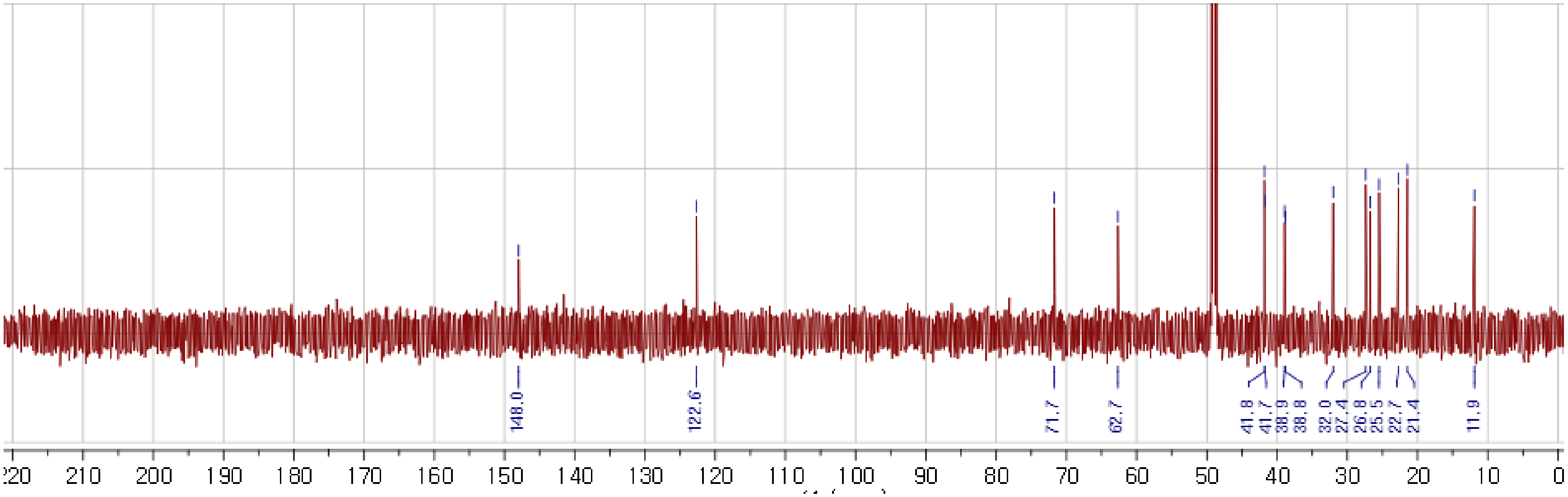

Figure S27. ^{13}C NMR spectrum of **9**.

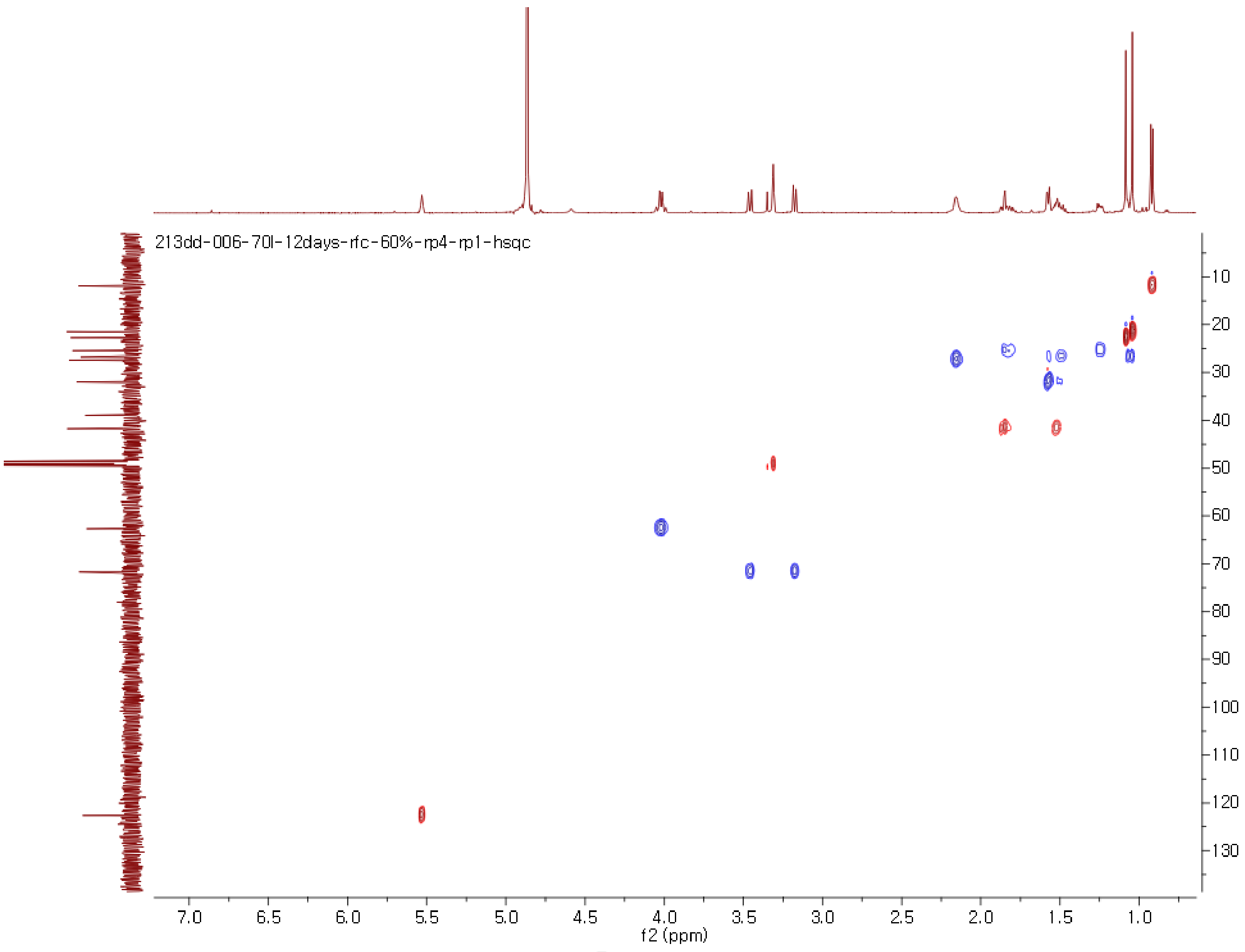

Figure S28. HSQC spectrum of **9**.

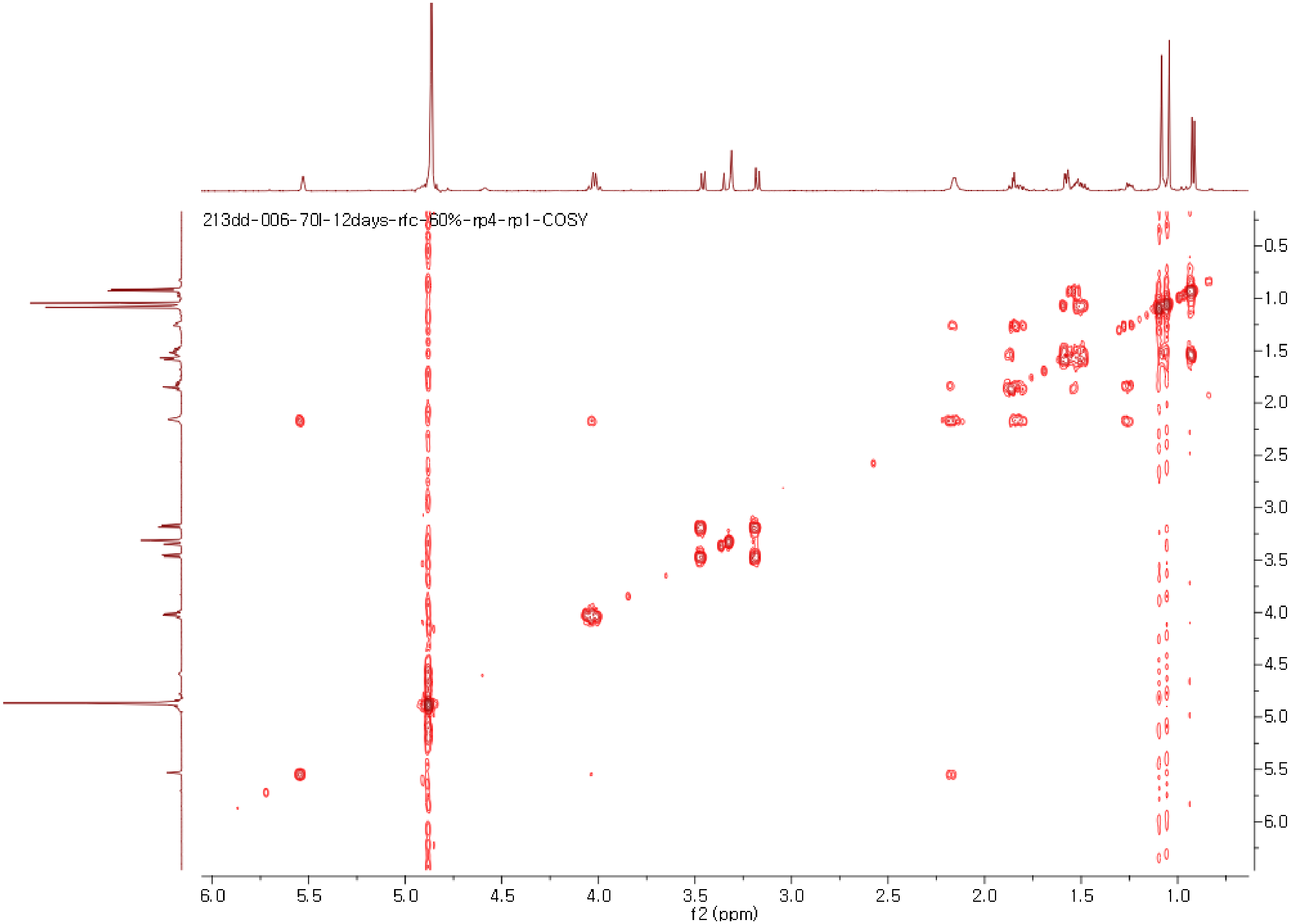

Figure S29. ^{1}H-^{1}H COSY spectrum of **9**.

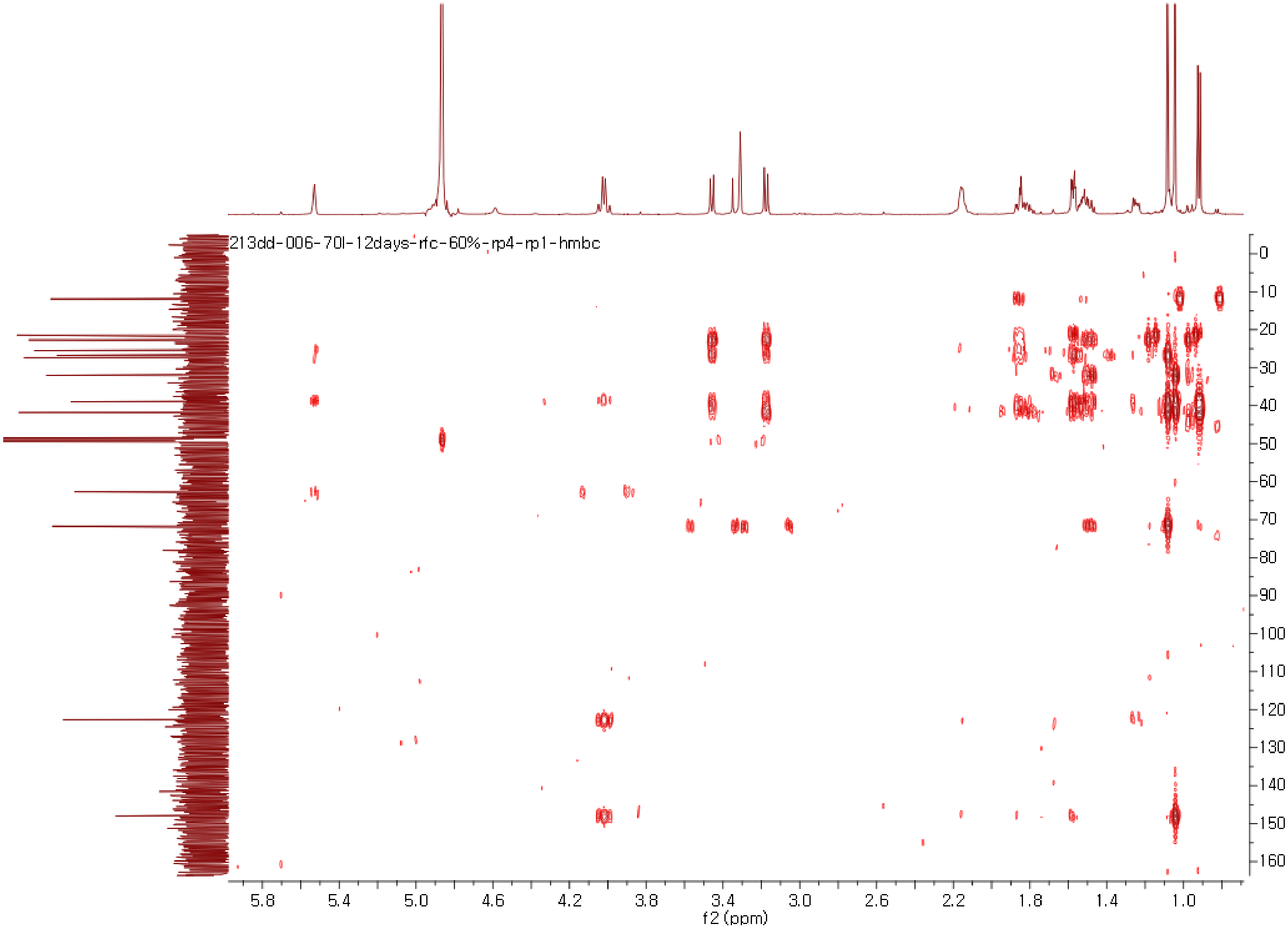

Figure S30. HMBC spectrum of **9**.

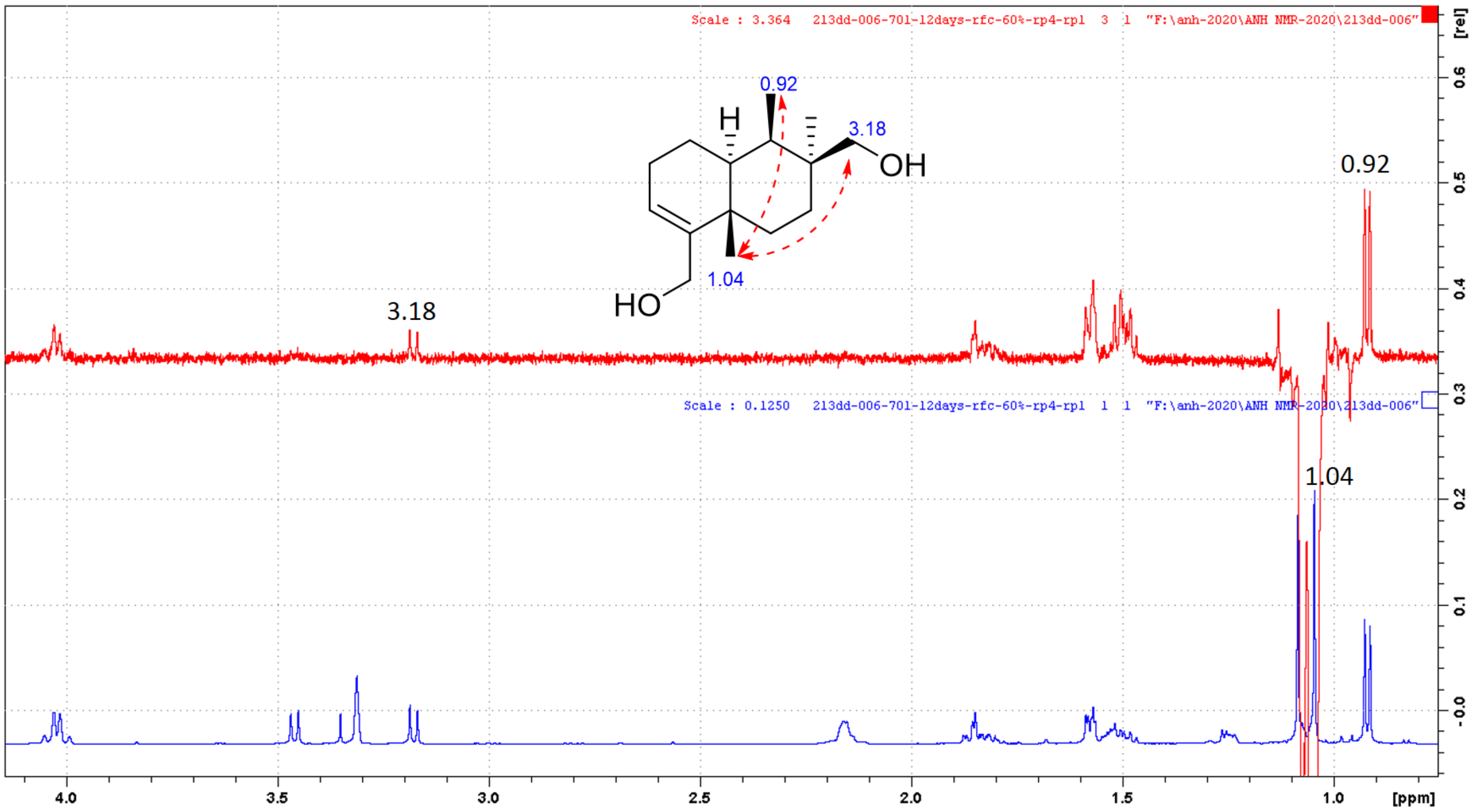

Figure S31. Selective 1D NOESY spectrum of **9** (irradiated at H_3-12).

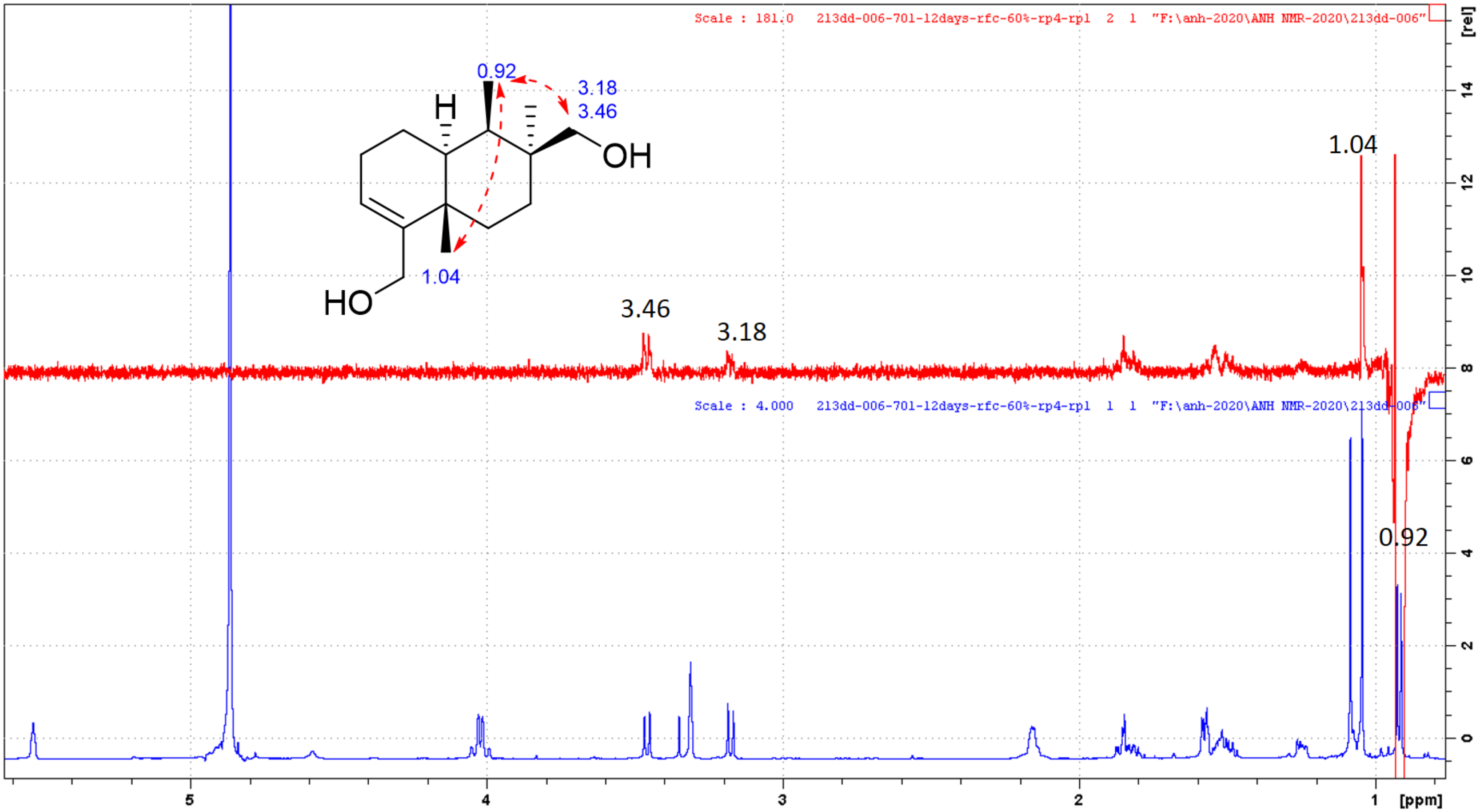

Figure S32. Selective 1D NOESY spectrum of **9** (irradiated at H_3-14).

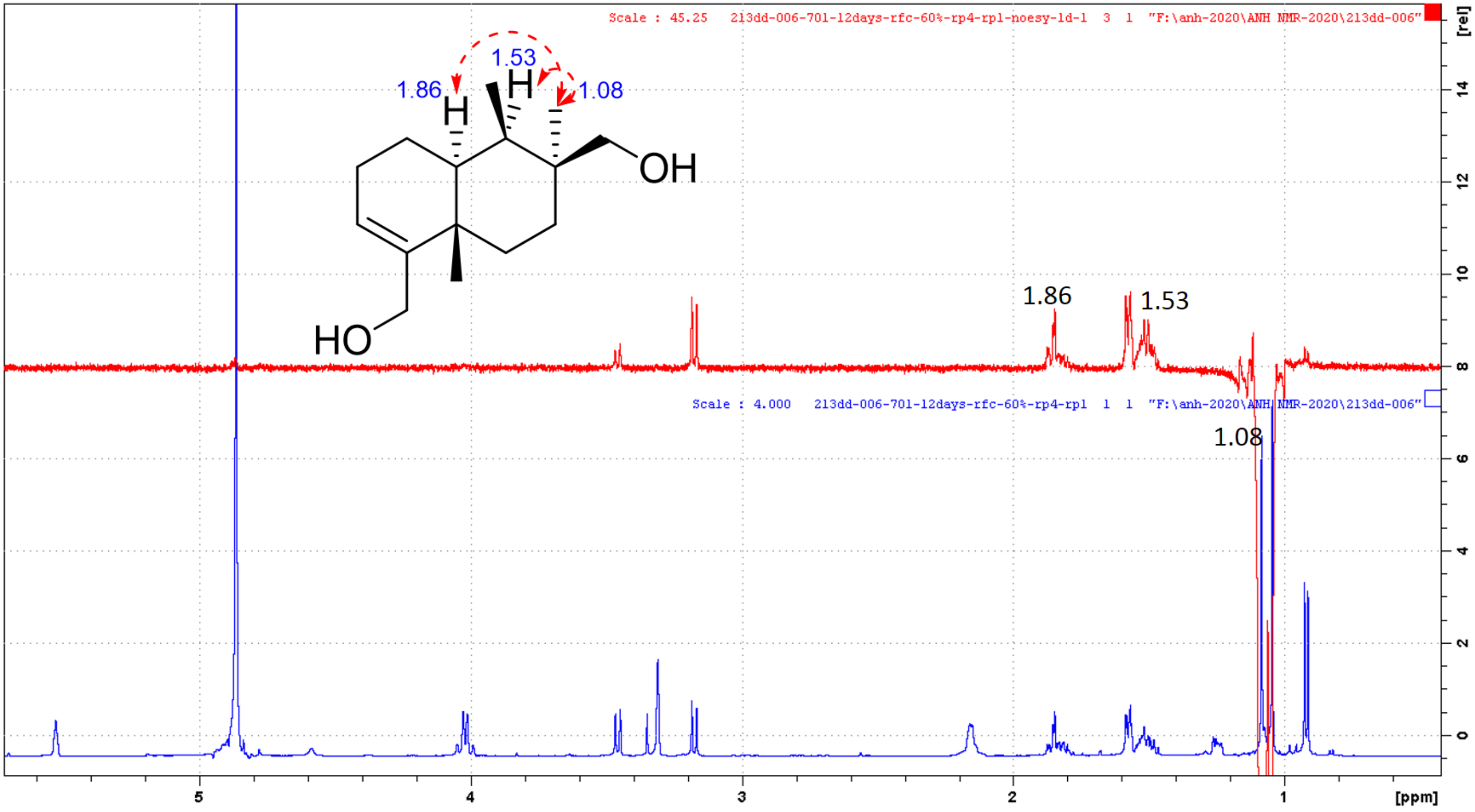

Figure S33. Selective 1D NOESY spectrum of **9** (irradiated at H_3-13).

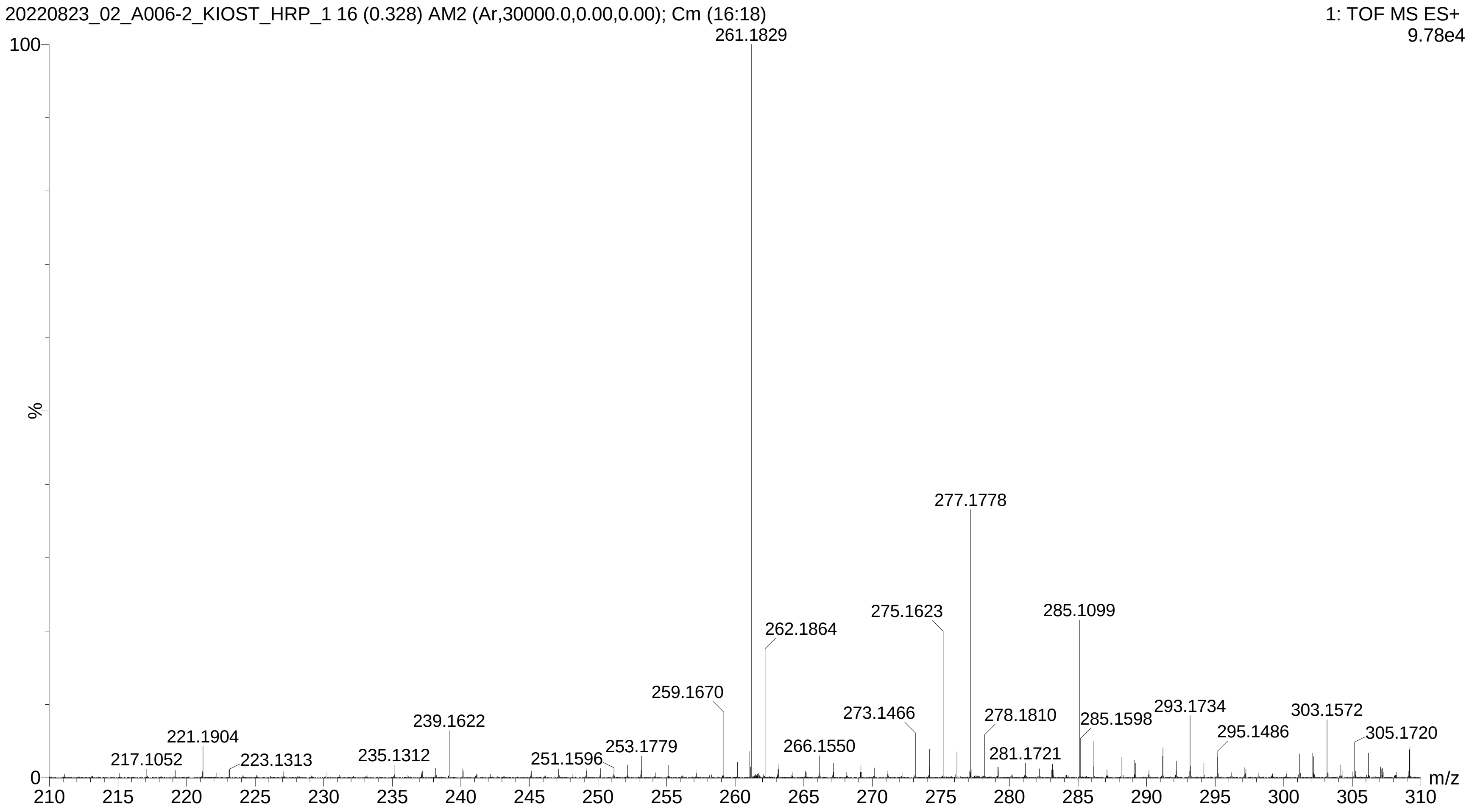

Mass	Calc. Mass	mDa	PPM	DBE	i-FIT	Norm	Conf(%)	Formula
261.1829	261.1830	-0.1	-0.4	2.5	941.8	n/a	n/a	C15 H26 O2 Na

Figure S34. HRESIMS data of **9**.

ACHN

Conc.(uM)	1 Mean	1 SD	2 Mean	2 SD	3 Mean	3 SD	4 Mean	4 SD	5 Mean	5 SD
30	80.52	6.02	86.30	4.34	75.45	5.45	-15.53	3.40	-9.47	7.91
10	88.42	5.74	90.30	7.11	84.29	3.31	27.38	6.75	21.55	5.26
3	90.11	5.84	94.05	3.29	92.49	6.33	32.37	7.47	28.72	9.93
1	89.92	2.70	97.44	1.06	95.10	5.56	87.50	4.89	84.26	5.07
0.3	94.85	5.54	98.75	1.74	97.70	2.30	95.35	2.98	96.94	3.86
GI50	>30		>30		>30		2.440	0.268	2.147	0.165

Conc.(uM)	6 Mean	6 SD	7 Mean	7 SD	8 Mean	8 SD	9 Mean	9 SD	Conc.	Adriamycin Mean	Adriamycin SD
30	-15.49	5.66	80.20	9.95	-14.66	2.09	76.28	7.21	3	-11.31	3.07
10	17.68	8.87	82.40	2.17	18.21	3.29	81.43	5.77	1	-6.82	8.81
3	40.66	4.80	88.08	6.96	25.64	7.09	84.05	4.43	0.3	13.78	6.87
1	76.40	8.49	95.05	5.64	82.71	5.44	95.54	3.76	0.1	44.22	6.42
0.3	92.41	3.53	96.49	4.19	96.99	3.01	97.60	5.20	0.03	97.10	4.53
GI50	2.309	0.060	>30		1.971	0.134	>30			0.095	0.005

MDA-MB-231

Conc.(uM)	1 Mean	1 SD	2 Mean	2 SD	3 Mean	3 SD	4 Mean	4 SD	5 Mean	5 SD
30	81.19	8.29	72.09	7.13	73.48	6.08	-12.50	4.57	-10.30	7.38
10	83.22	4.89	81.17	6.70	79.99	4.17	24.82	5.92	25.47	5.81
3	86.02	2.26	83.20	5.66	81.53	4.97	48.39	8.77	39.53	9.02
1	89.60	8.00	93.46	3.62	94.81	4.24	87.62	6.87	93.23	4.32
0.3	97.66	3.67	97.26	2.52	99.27	2.67	99.41	4.94	96.27	4.18
GI50	>30		>30		>30		3.216	0.151	2.881	0.392

Conc.(uM)	6 Mean	6 SD	7 Mean	7 SD	8 Mean	8 SD	9 Mean	9 SD	Conc.	Adriamycin Mean	Adriamycin SD
30	-10.50	7.67	77.49	4.20	-7.43	5.87	79.12	6.46	3	-12.39	3.45
10	27.33	5.27	79.99	5.58	25.97	8.37	81.53	6.44	1	-8.27	4.44
3	35.14	9.69	83.77	2.36	37.23	8.00	86.30	5.87	0.3	12.23	2.87
1	88.92	6.57	95.10	5.67	88.29	3.60	92.97	6.17	0.1	30.05	6.53
0.3	96.99	4.91	99.27	6.20	97.11	3.87	94.07	3.42	0.03	91.30	6.07
GI50	2.634	0.153	>30		2.712	0.101	>30			0.073	0.009

HCT-15

Conc.(uM)	1 Mean	1 SD	2 Mean	2 SD	3 Mean	3 SD	4 Mean	4 SD	5 Mean	5 SD
30	75.22	5.13	78.99	2.23	78.41	2.59	-9.93	2.66	-14.13	5.87
10	79.63	6.81	83.45	4.03	82.41	7.90	22.07	7.66	21.64	3.61
3	85.37	5.69	85.04	2.84	89.77	2.08	46.36	2.88	35.99	4.76
1	91.41	3.60	93.16	7.06	91.90	3.87	91.65	5.11	86.34	3.05
0.3	94.66	3.07	95.56	1.44	93.45	4.22	95.34	3.59	92.74	4.53
GI50	>30		>30		>30		3.115	0.017	2.458	0.349

Conc.(uM)	6 Mean	6 SD	7 Mean	7 SD	8 Mean	8 SD	9 Mean	9 SD
30	-4.90	4.06	81.42	3.53	-13.19	7.77	70.39	2.95
10	25.15	6.52	85.08	1.29	28.71	3.67	73.27	3.77
3	29.70	5.22	87.83	3.41	47.25	7.44	78.95	3.74
1	88.47	4.33	91.85	2.63	87.29	4.99	90.15	7.52
0.3	93.84	3.97	98.98	2.73	94.25	3.96	95.51	2.51
GI50	2.346	0.106	>30		3.233	0.122	>30	

	Adriamycin Mean	Adriamycin SD
3	-13.69	3.13
1	-5.25	4.38
0.3	13.77	5.34
0.1	34.67	4.26
0.03	94.62	7.61
	0.080	0.007

PC-3

Conc.(uM)	1 Mean	1 SD	2 Mean	2 SD	3 Mean	3 SD	4 Mean	4 SD	5 Mean	5 SD
30	84.93	3.15	72.30	5.51	82.88	1.55	-12.50	5.51	-7.53	8.94
10	87.14	4.46	78.41	2.61	84.31	3.75	23.38	5.68	24.11	4.35
3	88.60	5.71	87.30	3.00	89.37	3.72	40.99	3.59	52.64	2.76
1	94.72	2.10	93.38	4.97	94.25	4.25	93.38	1.74	87.00	4.84
0.3	99.45	1.21	96.67	3.49	99.19	3.03	96.28	1.08	96.87	4.22
GI50	>30		>30		>30		2.881	0.059	3.456	0.028

Conc.(uM)	6 Mean	6 SD	7 Mean	7 SD	8 Mean	8 SD	9 Mean	9 SD
30	-5.34	4.17	80.62	7.20	-5.68	7.86	81.49	4.94
10	20.57	6.68	85.78	5.48	24.60	3.92	82.34	7.30
3	43.24	5.02	87.91	6.76	45.51	5.62	89.68	2.86
1	90.56	3.71	93.53	5.05	93.51	2.55	93.11	4.82
0.3	97.58	3.53	99.79	1.99	98.86	3.21	99.34	2.97
GI50	2.922	0.197	>30		3.236	0.582	>30	

	Adriamycin Mean	Adriamycin SD
3	-11.65	4.66
1	-6.62	7.00
0.3	17.99	7.19
0.1	35.47	4.32
0.03	96.53	5.36
	0.084	0.010

NUGC-3

	1		2		3		4		5	
Conc.(uM)	Mean	SD	Mean	SD	Mean	SD	Mean	SD	Mean	SD
30	77.48	6.44	76.40	2.31	78.43	8.75	-20.18	3.09	-12.07	5.27
10	87.27	6.11	80.23	7.66	82.62	6.42	11.36	7.08	14.67	7.66
3	89.23	6.61	87.05	2.36	91.68	6.55	31.07	4.88	31.60	6.31
1	90.78	2.03	91.02	4.65	96.18	2.97	89.63	2.92	95.52	9.72
0.3	95.57	2.20	94.27	5.31	99.28	7.63	96.09	2.58	98.86	4.19
GI50	>30		>30		>30		2.234	0.135	2.368	0.383

	6		7		8		9			Adriamycin	
Conc.(uM)	Mean	SD	Mean	SD	Mean	SD	Mean	SD		Mean	SD
30	-8.42	2.55	82.57	4.81	-18.34	7.00	70.81	5.07	3	-15.59	7.40
10	13.07	7.03	85.95	4.77	15.04	8.53	72.09	3.98	1	-2.55	4.67
3	35.22	4.33	88.30	4.96	25.34	7.95	82.43	5.71	0.3	28.07	7.70
1	91.03	4.65	92.07	5.70	84.31	8.88	86.34	3.74	0.1	35.49	5.15
0.3	96.40	4.95	96.76	2.18	93.01	5.49	93.12	7.63	0.03	97.02	6.10
GI50	2.409	0.089	>30		1.967	0.440	>30			0.092	0.023

NCI-H23

	1		2		3		4		5	
Conc.(uM)	Mean	SD	Mean	SD	Mean	SD	Mean	SD	Mean	SD
30	76.80	4.89	73.14	4.54	73.85	0.96	-14.64	9.83	-8.90	6.07
10	81.76	7.02	79.10	5.11	76.12	8.47	28.14	7.29	16.77	8.70
3	85.83	2.86	86.06	3.19	80.37	2.28	36.22	8.83	43.09	9.58
1	89.55	3.71	92.21	6.06	88.42	4.70	86.38	5.09	87.61	8.02
0.3	97.25	4.84	93.73	2.38	93.40	2.32	92.56	6.87	93.49	4.00
GI50	>30		>30		>30		2.604	0.017	2.734	0.349

	6		7		8		9			Adriamycin	
Conc.(uM)	Mean	SD	Mean	SD	Mean	SD	Mean	SD		Mean	SD
30	-9.09	8.98	76.29	9.64	-16.11	3.14	80.30	2.67	3	-12.52	5.33
10	19.19	5.88	79.37	5.42	26.41	6.72	84.03	6.10	1	-9.96	4.52
3	41.82	5.56	83.43	5.24	31.14	8.27	86.21	7.76	0.3	17.71	6.52
1	88.79	7.43	89.94	9.52	90.31	3.09	87.18	5.35	0.1	25.15	8.75
0.3	94.67	2.52	93.73	7.45	95.45	1.71	98.14	1.61	0.03	93.70	4.84
GI50	2.753	0.106	>30		2.401	0.122	>30			0.070	0.007

Figure S35. Results of the cytotoxicity test of **1-9** against six solid cancer cell lines.

1	HL-60		Raji		K562		RPMI-8402		NALM6		U266		WSU-DLCL2	
(µM)	MEAN	SD	MEAN	SD	MEAN	SD	MEAN	SD	MEAN	SD	MEAN	SD	MEAN	SD
30	91.3	2.7	90.7	3.3	64.4	2.1	69.5	3.6	76.6	3.9	93.5	4.9	103.1	3.9
10	96.9	2.6	98.4	6.6	82.1	2.9	79.8	4.8	94.5	6.8	91.6	6.9	107.1	4.9
3	99.9	4.7	93.8	7.2	90.4	6.4	91.2	3.8	99.7	4.0	88.8	3.0	112.5	2.4
1	94.2	5.2	96.6	4.3	94.6	4.5	86.3	5.4	103.7	3.7	86.5	3.2	111.6	5.5
0.3	91.8	3.5	95.3	5.9	91.7	3.4	90.7	4.6	100.5	4.5	82.0	4.3	101.6	6.2
IC50	>30		>30		>30		>30		>30		>30		>30	

2	HL-60		Raji		K562		RPMI-8402		NALM6		U266		WSU-DLCL2	
(µM)	MEAN	SD	MEAN	SD	MEAN	SD	MEAN	SD	MEAN	SD	MEAN	SD	MEAN	SD
30	99.0	2.2	99.7	3.0	82.6	3.8	93.2	5.4	94.8	3.3	92.5	7.1	87.7	2.2
10	94.3	2.9	103.0	3.6	85.4	2.0	93.5	6.5	98.4	3.9	90.2	6.1	94.3	3.2
3	94.0	4.2	105.4	4.4	89.6	4.4	99.2	4.6	103.6	6.3	96.1	6.8	98.0	3.6
1	95.8	2.4	106.5	4.0	97.5	7.3	96.3	3.2	108.7	2.9	99.5	7.5	103.0	4.6
0.3	92.1	2.7	102.7	3.5	103.2	4.9	97.8	5.1	113.8	4.8	101.1	8.7	103.7	4.5
IC50	>30		>30		>30		>30		>30		>30		>30	

3	HL-60		Raji		K562		RPMI-8402		NALM6		U266		WSU-DLCL2	
(µM)	MEAN	SD	MEAN	SD	MEAN	SD	MEAN	SD	MEAN	SD	MEAN	SD	MEAN	SD
30	8.5	0.6	87.4	3.9	71.8	5.6	70.5	2.8	69.6	6.6	86.2	6.5	95.9	1.2
10	87.4	10.4	98.6	3.6	91.0	3.2	91.9	2.3	88.4	6.4	87.5	7.2	107.0	3.0
3	104.7	10.3	99.9	2.9	100.9	3.9	94.7	3.0	88.9	8.2	94.8	3.1	105.4	2.8
1	103.4	6.7	98.8	1.2	101.0	3.2	99.3	2.4	86.6	5.9	90.9	5.1	106.5	5.6
0.3	106.1	10.2	97.3	2.4	104.3	4.7	92.5	6.6	86.2	9.4	81.7	9.1	105.9	2.8
IC50	16.39	2.38	>30		>30		>30		>30		>30		>30	

4	HL-60		Raji		K562		RPMI-8402		NALM6		U266		WSU-DLCL2	
(μM)	MEAN	SD	MEAN	SD	MEAN	SD	MEAN	SD	MEAN	SD	MEAN	SD	MEAN	SD
30	94.9	2.4	82.6	8.4	36.0	1.1	38.1	1.1	9.7	1.8	99.1	2.7	69.1	5.7
10	94.9	2.9	120.3	7.8	109.7	5.0	97.6	2.7	92.1	7.1	104.9	1.6	105.6	0.9
3	91.3	4.3	105.7	4.4	109.5	6.2	96.3	2.2	84.8	8.3	102.9	6.6	104.4	2.8
1	94.3	1.0	102.7	5.7	112.8	6.1	91.6	1.5	92.2	3.5	105.1	3.1	93.2	2.8
0.3	92.0	4.0	98.0	5.5	113.9	3.8	92.4	2.6	85.2	5.5	108.5	6.5	84.5	2.9
IC50	>30		>30		28.23	0.85	26.39	0.63	17.75	1.73	>30		>30	

5	HL-60		Raji		K562		RPMI-8402		NALM6		U266		WSU-DLCL2	
(μM)	MEAN	SD	MEAN	SD	MEAN	SD	MEAN	SD	MEAN	SD	MEAN	SD	MEAN	SD
30	85.4	8.0	94.0	3.0	77.0	1.8	83.2	1.6	88.3	10.8	93.5	5.9	104.7	5.8
10	93.3	8.2	98.9	4.1	88.0	2.8	95.1	2.0	104.3	3.8	98.5	1.9	106.8	5.4
3	95.1	7.1	98.9	2.0	94.7	6.3	107.9	3.3	112.9	6.3	100.2	8.8	104.0	3.3
1	90.7	9.0	99.4	2.4	100.5	5.8	108.1	1.0	107.1	3.4	104.3	8.2	103.3	1.9
0.3	96.2	8.3	94.0	3.0	107.5	6.8	104.0	5.0	112.1	6.6	105.7	5.8	98.6	2.3
IC50	>30		>30		>30		>30		>30		>30		>30	

6	HL-60		Raji		K562		RPMI-8402		NALM6		U266		WSU-DLCL2	
(μM)	MEAN	SD	MEAN	SD	MEAN	SD	MEAN	SD	MEAN	SD	MEAN	SD	MEAN	SD
30	20.2	4.1	10.3	0.8	10.2	0.2	13.1	1.0	0.9	0.2	67.6	1.8	0.5	0.1
10	98.1	1.9	99.2	3.4	32.7	1.2	41.6	1.5	56.3	6.0	99.7	7.2	91.4	4.9
3	100.2	3.9	108.0	2.5	101.5	1.5	92.8	5.1	97.7	4.4	95.1	6.5	104.2	3.4
1	98.0	4.9	105.1	2.2	102.4	3.7	91.9	3.5	105.9	4.5	89.6	5.3	106.5	3.1
0.3	92.6	7.2	106.1	6.1	98.4	4.1	90.4	5.0	107.0	4.6	96.1	11.2	102.2	2.8
IC50	22.58	0.74	21.30	3.88	8.37	0.26	8.85	0.43	10.65	0.74	>30		14.03	1.17

7	HL-60		Raji		K562		RPMI-8402		NALM6		U266		WSU-DLCL2	
(μM)	MEAN	SD	MEAN	SD	MEAN	SD	MEAN	SD	MEAN	SD	MEAN	SD	MEAN	SD
30	98.7	3.9	120.4	3.0	98.6	5.0	100.2	2.3	88.9	4.3	99.5	4.2	107.9	2.6
10	98.3	4.6	111.9	2.2	106.8	7.9	97.5	2.4	96.7	4.8	101.8	5.2	108.9	2.5
3	90.8	2.2	111.7	4.0	98.3	8.5	96.9	4.0	87.1	7.5	103.2	2.5	105.7	2.8
1	96.1	3.5	109.3	7.9	100.8	9.8	90.9	5.1	97.6	4.0	103.1	2.9	94.6	6.9
0.3	91.6	2.3	101.1	8.0	102.8	6.0	88.0	4.9	99.9	5.8	101.5	6.9	87.1	6.9
IC50	>30		>30		>30		>30		>30		>30		>30	

8	HL-60		Raji		K562		RPMI-8402		NALM6		U266		WSU-DLCL2	
(μM)	MEAN	SD	MEAN	SD	MEAN	SD	MEAN	SD	MEAN	SD	MEAN	SD	MEAN	SD
30	83.6	4.5	52.8	2.0	17.7	3.9	27.4	1.6	8.7	0.4	90.6	3.0	36.8	8.4
10	93.1	3.8	98.3	3.9	85.1	0.7	99.5	5.9	109.3	7.5	103.9	9.3	100.5	6.2
3	93.4	3.6	98.1	3.7	100.5	5.0	107.4	6.3	93.7	3.2	101.4	4.5	97.5	4.6
1	92.5	3.0	95.7	4.8	99.2	6.6	105.4	6.5	93.9	10.6	100.7	6.4	98.9	2.7
0.3	93.7	1.1	94.3	4.4	102.0	3.5	106.2	4.6	96.8	7.7	97.1	7.4	100.1	1.2
IC50	>30		>30		17.94	1.05	25.37	2.90	24.77	1.22	>30		28.26	3.45

9	HL-60		Raji		K562		RPMI-8402		NALM6		U266		WSU-DLCL2	
(μM)	MEAN	SD	MEAN	SD	MEAN	SD	MEAN	SD	MEAN	SD	MEAN	SD	MEAN	SD
30	94.6	2.6	108.5	11.6	91.8	2.8	88.2	5.4	89.1	3.2	97.8	6.3	94.2	2.7
10	92.6	6.6	112.0	13.7	96.6	10.1	86.6	4.2	93.6	1.8	96.5	6.2	105.5	3.7
3	95.5	5.4	110.2	8.0	101.8	7.9	90.2	5.9	117.1	7.1	89.0	7.5	113.5	2.4
1	99.3	4.9	94.2	9.2	100.6	7.5	89.9	2.1	128.9	13.9	89.1	5.4	104.5	8.0
0.3	96.7	6.1	89.4	11.8	96.0	6.4	92.8	5.2	97.6	1.0	81.1	2.5	94.1	8.7
IC50	>30		>30		>30		>30		>30		>30		>30	

Doxorubicin HCl	HL-60		Raji		K-562		RPMI-8402		NALM6		U266		WSU-DLCL2	
(μM)	MEAN	SD	MEAN	SD	MEAN	SD	MEAN	SD	MEAN	SD	MEAN	SD	MEAN	SD
1	0.1	0.1	0.6	0.1	5.8	0.6	2.0	0.2	1.1	0.1	1.9	0.1	0.0	0.1
0.3	0.1	0.1	0.3	0.1	6.1	0.8	0.3	0.1	0.1	0.1	0.7	0.1	0.0	0.1
0.1	1.0	0.2	0.2	0.1	49.3	2.2	8.3	1.4	0.0	0.1	4.7	0.5	0.2	0.1
0.03	30.7	0.9	4.8	1.0	83.0	2.4	31.0	1.2	-0.1	0.0	60.3	3.2	3.1	0.3
0.01	69.3	1.5	46.6	5.5	97.8	2.7	69.3	2.4	0.4	0.1	96.3	4.3	14.5	1.4
0.003	90.8	1.7	75.6	3.8	100.3	3.7	85.6	5.1	57.6	4.7	81.2	3.4	56.7	1.7
0.001	96.9	1.4	102.9	2.2	102.3	4.5	101.9	6.5	100.0	5.4	93.1	3.6	95.0	4.9
0.0003	98.8	1.6	114.0	4.3	105.3	8.4	101.6	3.1	101.4	5.6	91.6	7.8	101.1	6.0
0.0001	102.3	5.2	116.1	2.9	115.3	3.7	103.0	6.6	100.0	5.8	95.3	5.2	102.1	3.0
IC50	0.0167	0.0010	0.0080	0.0010	0.0901	0.0020	0.0169	0.0000	0.0032	0.0000	0.0349	0.0010	0.0036	0.0000

Figure S36. Results of the cytotoxicity test of **1-9** against seven blood cancer cell lines.
